# Theabrownin from Pu-erh tea attenuates hypercholesterolemia via modulation of gut microbiota and bile acid metabolism

**DOI:** 10.1038/s41467-019-12896-x

**Published:** 2019-10-31

**Authors:** Fengjie Huang, Xiaojiao Zheng, Xiaohui Ma, Runqiu Jiang, Wangyi Zhou, Shuiping Zhou, Yunjing Zhang, Sha Lei, Shouli Wang, Junliang Kuang, Xiaolong Han, Meilin Wei, Yijun You, Mengci Li, Yitao Li, Dandan Liang, Jiajian Liu, Tianlu Chen, Chao Yan, Runmin Wei, Cynthia Rajani, Chengxing Shen, Guoxiang Xie, Zhaoxiang Bian, Houkai Li, Aihua Zhao, Wei Jia

**Affiliations:** 1 Shanghai Key Laboratory of Diabetes Mellitus and Center for Translational Medicine, Shanghai Jiao Tong University Affiliated Sixth People’s Hospital, Shanghai, China; 2 School of Pharmacy, Shanghai Jiao Tong University, Shanghai, China; 3 Department of Pharmacology and Toxicology, Tasly Pharmaceutical Co. Ltd, Tianjin, China; 4 University of Hawaii Cancer Center, Honolulu, USA; 5 Department of Cardiology, Shanghai Jiao Tong University Affiliated Six People’s Hospital, Shanghai, China; 6 Chinese Medicine Clinical Study Center, School of Chinese Medicine, Hong Kong Baptist University, Hong Kong SAR, China; 7 Functional Metabolomic and Gut Microbiome Laboratory, Institute of Interdisciplinary Integrative Medicine Research, Shanghai University of Traditional Chinese Medicine, Shanghai, China

**Keywords:** Microbiome, Fat metabolism, Metabolic diseases, Microbiota

## Abstract

Pu-erh tea displays cholesterol-lowering properties, but the underlying mechanism has not been elucidated. Theabrownin is one of the most active and abundant pigments in Pu-erh tea. Here, we show that theabrownin alters the gut microbiota in mice and humans, predominantly suppressing microbes associated with bile-salt hydrolase (BSH) activity. Theabrownin increases the levels of ileal conjugated bile acids (BAs) which, in turn, inhibit the intestinal FXR-FGF15 signaling pathway, resulting in increased hepatic production and fecal excretion of BAs, reduced hepatic cholesterol, and decreased lipogenesis. The inhibition of intestinal FXR-FGF15 signaling is accompanied by increased gene expression of enzymes in the alternative BA synthetic pathway, production of hepatic chenodeoxycholic acid, activation of hepatic FXR, and hepatic lipolysis. Our results shed light into the mechanisms behind the cholesterol- and lipid-lowering effects of Pu-erh tea, and suggest that decreased intestinal BSH microbes and/or decreased FXR-FGF15 signaling may be potential anti-hypercholesterolemia and anti-hyperlipidemia therapies.

## Abstract (web summary)

Pu-erh tea displays cholesterol-lowering properties. Here, Huang et al. show that this is mostly due to the action of a pigment in Pu-erh tea that induces changes in certain gut microbiota and bile acid levels, thus modulating the gut-liver metabolic axis.

## Introduction

Pu-erh, a famous traditional Chinese tea exclusively produced in the Yunnan district, Southwest China through microbial fermentation of fresh *Camellia sinensis* leaves, has been reported to possess multiple beneficial effects including attenuation or reversal of hypercholesterolemia, hyperlipidemia, obesity, steatohepatitis, and hyperglycemia^1^. The anti-obesity and anti-hyperlipidemic effects have been well documented by numerous studies in which Pu-erh tea consumption reduced body weight, weight of adipose pads, serum and hepatic levels of total cholesterol (TC), total triglyceride (TG), and low-density lipoprotein-cholesterol (LDL-C) in rats, mice, and human subjects^2^. However, most of these studies were observational in nature and the underlying mechanisms for these effects have not been determined.

Comparative studies using rodents treated with Pu-erh tea, green tea, and black tea^3^ provided supporting evidence that fully fermented Pu-erh tea is more effective in causing hypolipidemic and hypocholesterolemic effects compared to other partially fermented and non-fermented teas. Therefore, we hypothesized that certain components generated in the unique Pu-erh tea fermentation process caused the observed stronger biological effects. The differences in the active compounds in green, black and Pu-erh teas have also been widely investigated^4^. A previous study done in our lab revealed that the characteristic components of the various teas were theaflavin and theanin in green tea; thearubigin and theaflavic acid in black tea; and theabrownin and gallic acid in Pu-erh tea^5^. During the fermentation process, the catechins and their gallate derivatives are oxidized to complex phenolic tea pigments including theaflavins (TF), thearubigins (TR) and, theabrownins (TB). Theaflavins undergo further oxidation to form the more polymerized thearubigins, which are then condensed to theabrownins^6^. To summarize, catechins, TF, and TR are reduced in concentration while TB is greatly increased during the Pu-erh tea fermentation process, indicating that theabrownin is a characteristic constituent of Pu-erh tea and thus, may be the bioactive substance responsible for its hypocholesterolemic and hypolipidemic effects.

Bile acids (BAs) are the dominant downstream products of cholesterol catabolism and therefore, the production and excretion of BAs is critical for the maintenance of cholesterol homeostasis. Farnesoid X receptor (FXR) is a BA-activated nuclear receptor that regulates the homeostasis of BAs, lipids and glucose^7,8^. Upon activation of intestinal FXR, the hormone, fibroblast growth factor 15 (FGF15) is produced, subsequently secreted into the portal vein and circulated to the liver where it binds to the fibroblast growth factor receptor 4 (FGFR4). The FGF15-FGFR4 complex initiates a signaling cascade that results in the inhibition of hepatic BA biosynthesis from cholesterol^9–11^. Emerging evidence suggested that inhibition of ileal FXR-FGF15 induced beneficial effects that can lead to the improvement of non-alcoholic fatty liver disease (NAFLD), obesity, and insulin resistance^12–14^. Further, it has been reported that T-βMCA, one of the primary BA produced in mice only, is a naturally occurring FXR antagonist^15^.

Gut microbiota have been found to play an important role in regulating enterohepatic BA metabolism via their ability to biotransform BAs into forms which have strong regulatory effects on BA signaling receptors^16,17^. Primary BAs are synthesized from cholesterol in the liver, conjugated with either glycine or taurine, and further metabolized by the gut microbiota into secondary BAs by undergoing a series of deconjugation, dehydrogenation, dehydroxylation, and isomerization processes. BAs can reshape the gut microbiota composition through direct modulation of the bile-sensitive and bile-metabolizing bacteria^18–20^, and also via FXR-mediated transcription of antimicrobial agents (e.g., iNOS and IL-18) that affect the gut microbiota via the immune system^21,22^. The gut microbiota-BA interaction plays a key role in regulating energy harvest, lipid metabolism as well as cholesterol and BA homeostasis^15,23,24^.

In this study, we find that theabrownin from Pu-erh tea suppresses the bile salt hydrolase (BSH) related microbes and BSH activity. Reduced BSH activity results in increased ileal conjugated BAs which further inhibit the intestinal FXR-FGF15/19 signaling pathway to elevate hepatic BA production. In the regulation of BA synthesis by theabrownin, intestinal FXR-FGF15/19 signaling is inhibited while hepatic FXR-SHP signaling is activated, resulting in the increased expression of enzymes in the alternative BA synthetic pathway, elevate hepatic BA production and fecal excretion and ultimately, reduced cholesterol level. The results of this study show a mechanistic link between theabrownin, the characteristic component of Pu-erh tea, and changes in the gut microbiota, FXR signaling and BA synthesis in the modulation of cholesterol levels in serum and liver.

## Results

### Pu-erh tea reduced HFD induced weight gain and hyperlipidemia

**Fig. 1:**
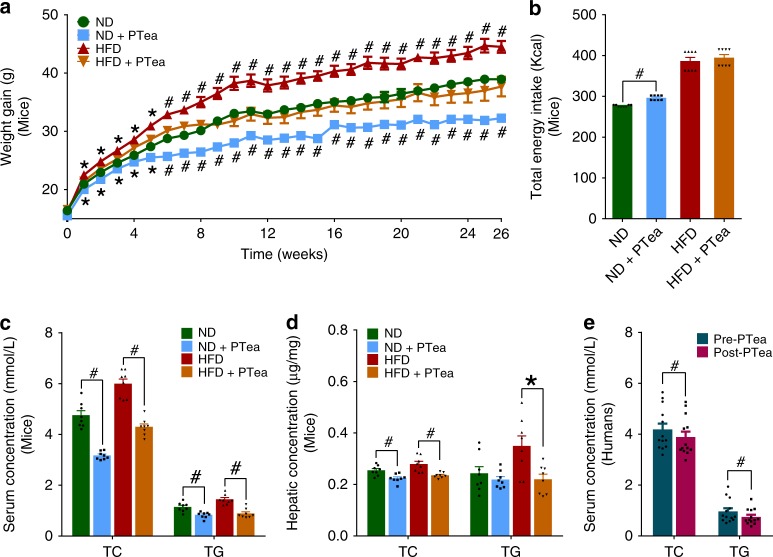
Body weight and lipid lowering effects of Pu-erh tea. **a** Pu-erh tea reduced the body weight of mice with either normal diet or HFD for 26 weeks. *n* = 8 individuals/group. Data in ND and HFD groups were compared to ND + PTea and HFD + PTea groups, respectively. **b** The total energy intake of normal diet or HFD fed mice after 26 weeks of tea intervention. *n* = 8 individuals/group. **c** Pu-erh tea reduced serum lipids of mice with either normal diet or HFD for 26 weeks. *n* = 8 individuals/group. **d** Pu-erh tea reduced hepatic TG and TC contents of mice with either normal diet or HFD. *n* = 8 individuals/group. **e** Pu-erh tea reduced serum lipids of human subjects after 4 weeks of consumption. *n* = 13 individuals/group. Data were expressed as mean ± SEM. Differences of data in mice and human subjects were assessed by the Mann–Whitney U test and Wilcoxon rank-sum test, respectively, *p < 0.05, ^#^*p* < 0.005

Instant Pu-erh tea is produced using a specific, standardized manufacturer’s protocol, which includes multi-stage countercurrent extraction and spray drying. The resulting tea components are more homogeneous and reproducible than those extracted from ripe Pu-erh tea. Therefore, instant Pu-erh tea (in the form of powder) was selected for use in our experiments. Male C57BL/6 J mice were fed with normal chow (ND group) and high fat diet (HFD group) for 26 weeks from 4 weeks of age. Half of the mice in each of the two groups received 3 mg/mL instant Pu-erh tea infusion in their water bottles (ND + PTea and HFD + PTea groups). Meanwhile, male human subjects were supplied with a standard diet for 1 week (baseline, Pre-PTea) and subsequently supplied with 300 mL of 5 mg/mL instant Pu-erh tea twice a day for 4 weeks (Post-PTea) (Supplementary Fig. 1). After 26 weeks of tea treatment, both ND and HFD mice exhibited significantly decreased body weights in the HFD + PTea group similar to those in the ND group (Fig. 1a). The total energy intake of all the mice for the 26-week experiment was measured (Fig. 1b), showing that ND + PTea and HFD + PTea mice consumed slightly higher energy amounts relative to ND and HFD mice, respectively, inferring that Pu-erh tea induced weight loss was not due to reduced energy intake. In the meanwhile, the drinking volume of Pu-erh tea in ND + PTea and HFD + PTea groups were more than the drinking volume of water in ND and HFD groups, respectively (Supplementary Fig. 2), indicating that Pu-erh tea exposure did not diminish the drinking habits of the mice. The body weight of human subjects was not decreased significantly during 4 weeks of 50 mg/Kg/day Pu-erh tea consumption on a standard diet (Supplementary Fig. 3).

Pu-erh tea consumption decreased the blood and hepatic lipids in mice revealed by a significantly lower level of TC and TG identified in the tea intervention groups (Fig. 1c, d). These changes in lipids induced by Pu-erh tea were confirmed by the reduction of serum TC and TG in human subjects (Fig. 1e). These results showed the preventive effect of the Pu-erh tea consumption on hyperlipidemia. In another study focused on the therapeutic effect of Pu-erh tea, we conducted experiments in which we treated HFD mice with Pu-erh tea at different time to verify the anti-obesity and anti-hyperlipidemic effects of Pu-erh tea in short, medium, or long term HFD. Mice fed HFD for 4, 22, and 42 weeks were treated with Pu-erh tea for 4 weeks. The results showed that the body weight, level of TC and TG were decreased by Pu-erh tea treatment (Supplementary Fig. 4a, b). Therefore, our results indicated that either 50 or 450 mg/Kg/day of Pu-erh tea powder consumption for human subjects and mice, respectively, could markedly prevent weight gain from HFD in mice and decrease hyperlipidemia in both humans and mice. This suggested that Pu-erh tea has a therapeutic effect on weight gain and serum TC and TG concentrations.

### Pu-erh tea reduced BSH enriched bacteria and BSH activity

**Fig. 2:**
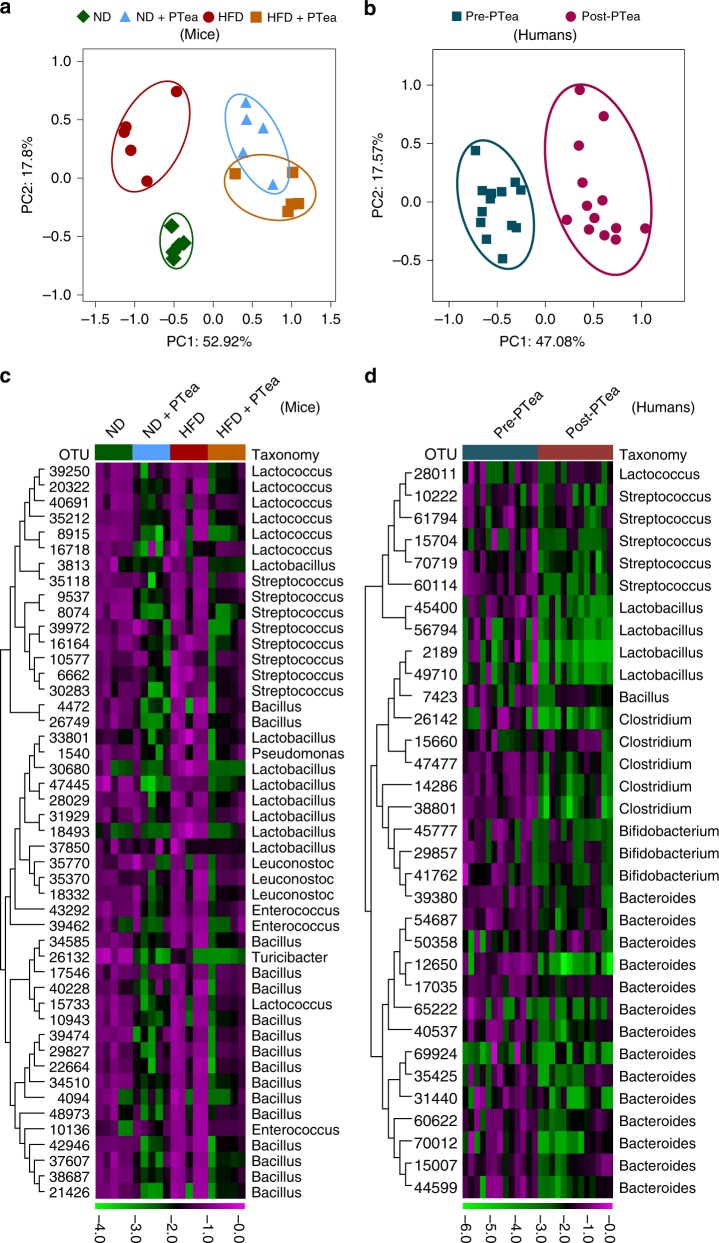
Pu-erh tea reshaped the gut microbiota in mice and human. **a** Principle coordinate analysis (PCoA) plot based on the OTU matrix of mouse ileal microbiota in ND, ND + PTea, HFD and HT + PTea groups. *n* = 5 individuals/group. **b** PCoA plot based on the OTU matrix of human fecal microbiota. *n* = 13 individuals/group. **c** Heatmap of the relative abundance of representative OTUs at the genus and species levels in mice. Part of the OTUs which were simultaneously reduced by Pu-erh tea in both normal diet and HFD are shown, which can be referenced to the OTUs in blue outline of the full figure (Supplemantary Fig. 6a). *n* = 5 individuals/group. **d** Heatmap of the relative abundance of representative OTUs at the genus and species levels in humans. *n* = 13 individuals/group. Part of the OTUs which were reduced by Pu-erh tea similar to mice are shown, which can be referenced to the OTUs in blue outline of the full figure (Supplemantary Fig. 6b). The color of each spot in the heatmap corresponds to the normalized and log-transformed raw abundance of the OTUs in each sample. The OTUs were organized according to their order in the phylogenetic tree generated by their representative sequences

The overall structural changes of gut microbiota in response to instant Pu-erh tea were determined by analysis of the 16S rRNA gene sequences of microbial samples isolated from the ileum of ND, ND + PTea, HFD and HFD + PTea mouse groups. UniFrac distance-based principal coordinate analysis (PCoA) revealed distinct clustering of intestinal microbe communities for each experimental group. Remarkable changes in the microbiota community structure were induced by both HFD and Pu-erh tea intervention. The microbes in ND + PTea and HFD + PTea groups were more closely clustered relative to ND and HFD groups, which is an indication that tea consumption induced similar microbial composition changes, particularly in two of the PTea control groups with respect to HFD + PTea (Fig. 2a). Pu-erh tea induced changes in the community structure of human fecal microbes as revealed by the PCoA plot (Fig. 2b). LEfSe analyses showed that common changes occurred in the relative abundance of different levels where the classes of Bacilli and α-Proteobacteria were reduced while the relative abundance of different levels within the class Bacteroidia were enriched in the ND + PTea and HFD + PTea groups (Supplementary Fig. 5a). Similarly, the relative abundances of the classes, Bacilli and Clostridia, were reduced in post-tea groups in human feces (Supplementary Fig. 5b). In general, Pu-erh tea-induced microbial changes in mice and human showed the same tendencies at the Phylum and Class levels. Some of the relative abundance changes at the genus level were also identified. OTUs in the *Lactobacillus, Bacillus, Enterococcus, Lactococcus, Streptococcus*, and *Leuconostoc* genera were reduced, showing the same differential tendencies in both the ND + PTea and HFD + PTea groups of mice (Fig. 2c and Supplementary Fig. 6a). In human stool samples, the microbiota changes at the genus level showed that the relative abundances of OTUs in *Lactobacillus, Bacillus, Streptococcus* and *Lactococcus* genera were reduced by tea intervention and thus resembled the changes seen in mice (Fig. 2d and Supplementary Fig. 6b).

**Fig. 3:**
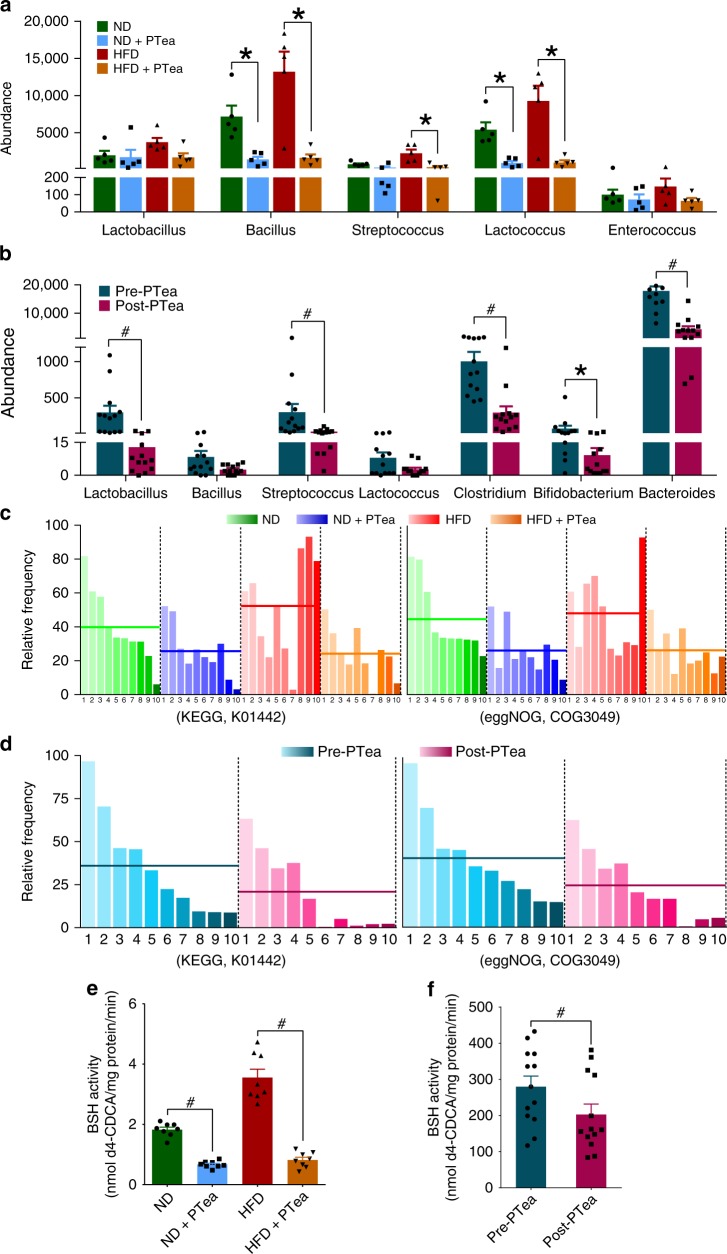
Pu-erh tea reduced the abundance of BSH microbes and BSH activity. **a** Abundance of BSH enriched microbes in ileum of mice undergoing Pu-erh tea consumption. *n* = 5 individuals/group. **b** Abundance of BSH enriched microbes in feces of humans consuming Pu-erh tea. *n* = 13 individuals/group. **c** The microbial BSH enzyme activity in the ileal contents of mice treated with Pu-erh tea. **d** The microbial BSH activity in human feces from subjects on Pu-erh tea consumption. The functional profiles were identified by metagenomic analysis utilizing both KEGG and eggNOG databases. BSH related proteins ranked in the top ten are shown and the mean abundances are illustrated as the horizontal line in each group. **e** Ileal BSH activity in mice on Pu-erh tea consumption. *n* = 8 individuals/group. **f** Fecal BSH activity inhuman subjects on Pu-erh tea consumption. *n* = 13 individuals/group. Data were expressed as mean ± SEM. Differences in the data for mice and human subjects were assessed by the Mann–Whitney U test and Wilcoxon rank-sum test, respectively, *p < 0.05, ^#^*p* < 0.005

The common function of these reduced microbial genera is to generate the BSH enzymes which are used to deconjugate glycine- or taurine-conjugated BAs to form unconjugated BAs. The reduction of BSH positive bacteria was evident in both mice and human subjects (Fig. 3a, b). In order to identify the microbial genes related functional pathways present in the small intestine, a metagenomic approach (the whole genome shotgun) was employed to produce a metabolic function profile. BSH activity was described mainly by the PVAs (EC 3.5.1.11) and CGH (EC 3.5.1.24) in KEGG and eggNOG databases, respectively. All the BSH related proteins identified in KEGG and NOG databases were reduced by instant Pu-erh tea and the decreased abundance of the BSH-related proteins in the top ten were shown in both mice and human subjects (Fig. 3c, d).

We measured BSH activity in the small intestine using a previously described method^25,26^. The results showed that BSH activity in the small intestine of both ND + PTea and HFD + PTea groups of mice as well as in stool samples of the post-tea group of human subjects was significantly attenuated (Fig. 3e, f).

### Pu-erh tea induced an accumulation of conjugated BAs in ileum

**Fig. 4:**
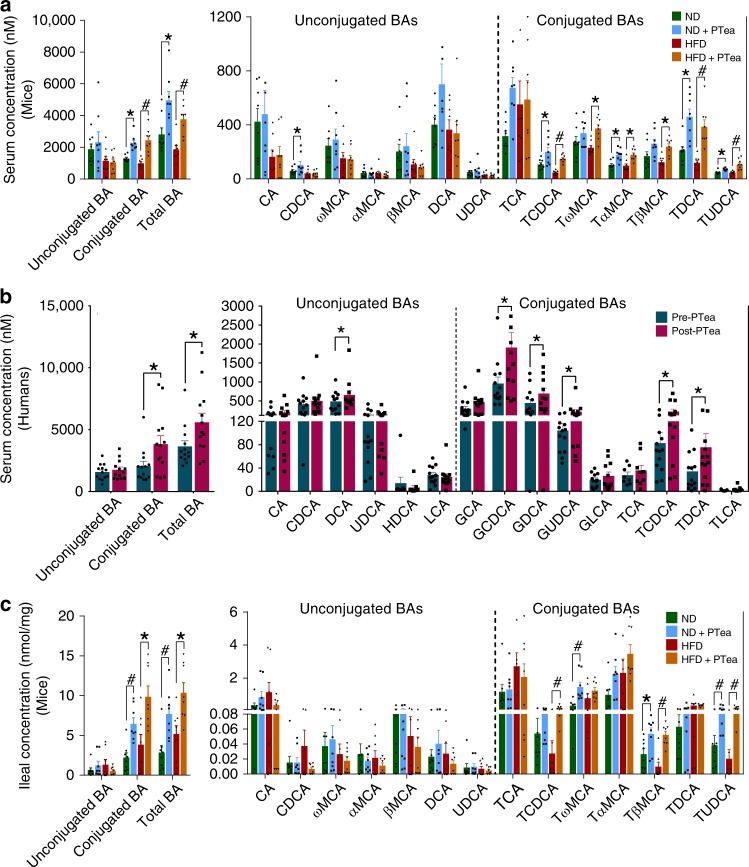
Pu-erh tea elevated conjugated BAs in serum and ileum. **a** Serum BA classes and BA profile of mice after 450 mg/Kg/day Pu-erh tea consumption for 26 weeks. *n* = 8 individuals/group. **b** Serum BA classes and BA profile of human subjects after 50 mg/Kg/day Pu-erh tea consumption for 4 weeks. *n* = 13 individuals/group. **c** Ileal BA classes and BA profile of mice after 450 mg/Kg/day Pu-erh tea consumption for 26 weeks. *n* = 8 individuals/group. Data were expressed as mean ± SEM. Differences of data for mice and human subjects were assessed using the Mann–Whitney U test and Wilcoxon rank-sum test, respectively, *p < 0.05, ^#^*p* < 0.005

To determine the effects of depleted BSH activity on the BA profile, a UPLC/TQMS (ultra-performance liquid chromatography triple-quadrupole mass spectrometry) based targeted metabolomics approach was used to analyze the BAs in the serum and feces of both mice and human subjects. The results revealed that the serum levels of tauro-conjugated BAs were dramatically elevated, most significantly, tauro-chendoxycholic acid (TCDCA) and tauro-ursodeoxycholic acid (TUDCA) in mice, and the glycine-conjugated BAs, glyco-chenodeoxycholic acid (GCDCA) and glycol-ursodeoxycholic acid (GUDCA), were significantly increased in human subjects (Fig. 4a, b). Similarly, the conjugated BAs, TCDCA, and TUDCA, in the distal ileum of mice were elevated in the Pu-erh tea treatment groups (Fig. 4c). Furthermore, fecal BA excretion was increased in Pu-erh tea treated mice and human subjects (Supplementary Fig. 7a, b). The changes in BA composition along with increased concentrations of conjugated BAs, further confirmed that Pu-erh tea decreased the BSH activity of intestinal microbiome.

### Theabrownin reduced BSH bacteria abundance and BSH activity

**Fig. 5:**
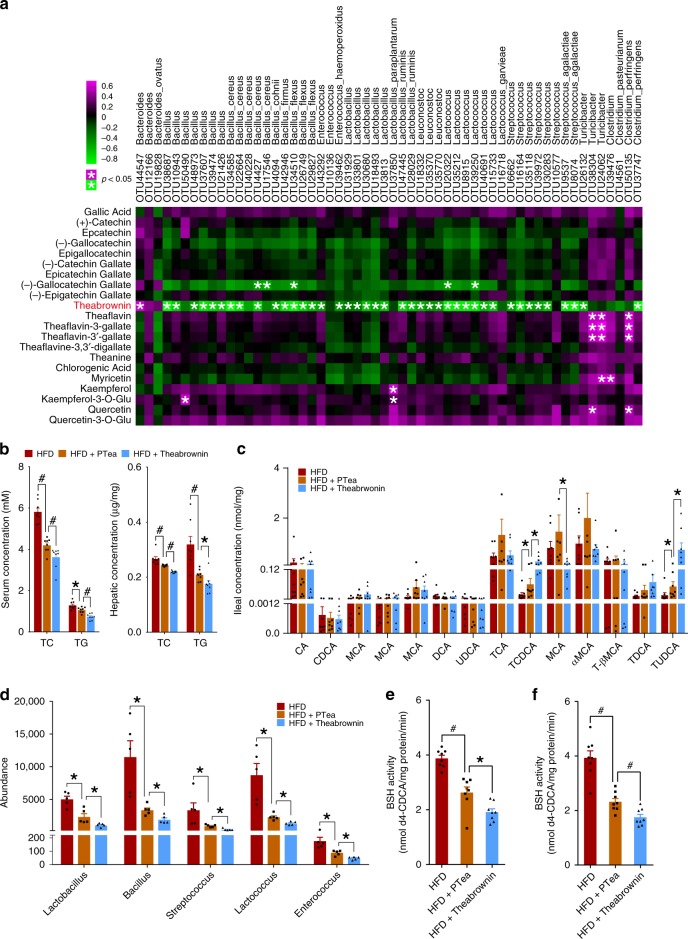
Theabrownin directly reduced BSH activity to elevate ileal conjugated BAs. **a** Spearman correlation between Pu-erh tea constituents and OTUs of BSH-producing microbes in mice fed normal diet and HFD supplied with 450 mg/Kg/day Pu-erh tea for 26 weeks. *n* = 8 individuals/group. The color of each spot in the heatmap corresponds to the R value of the spearman correlation analysis between microbial abundance and tea components concentration, and the spot with asterisk in magenta color refers to the significant positive correlation with *R* > 0.3 and *P* < 0.05 while the spot with asterisk in green color refers to the significant negative correlation with *R* < −0.3 and *P* < 0.05. **b** Pu-erh tea and theabrownin reduced serum and hepatic TC and TG of mice. *n* = 8 individuals/group. **c** Ileal BAs changes in Pu-erh tea and theabrownin intervention in vivo. *n* = 8 individuals/group. **d** BSH-producing microbe changes induced by Pu-erh tea and theabrownin in vivo. *n* = 8 individuals/group. **e** Ileal BSH activity changes induced by Pu-erh tea and theabrownin in vivo. *n* = 8 individuals/group. **f** Theabrownin and Pu-erh tea reduced BSH activity in cultured ileal microbes. *n* = 8 individuals/group. Data were expressed as mean ± SEM. Differences between data were assessed using the Mann–Whitney U test, *p < 0.05, ^#^*p* < 0.005

To identify the dominant compounds in instant Pu-erh tea responsible for reshaping the gut microbiota with suppressed BSH activity, we quantified the tea components including tea polyphenols and tea pigments in the ileal contents of mice by UPLC/QTOFMS (quadrupole-time-of-flight mass spectrometry). Of all the tea components, theabrownin showed the most significantly negative correlation with almost all the BSH-producing microbe OTUs (Fig. 5a). We further conducted metagenomic analysis of microbiota in ileum of the mice treated with theabrownin (extracted from Pu-erh tea, >95% of purity, Supplementary Fig. 8, see Material and Methods) for 8 weeks. The data showed that BSH function and the BSH related microbiota species were significantly decreased as a result of theabrownin treatment (Supplementary Fig. 9a, b). We therefore hypothesized that theabrownin was responsible for reducing ileal BSH-producing microbes and BSH activity.

To verify this hypothesis, mice fed HFD were treated with Pu-erh tea and theabrownin for 8 weeks, respectively. The changes in ileal BSH-producing microbes, BSH activity, levels of conjugated BAs, TC and TG in serum and liver were measured. As the concentration of theabrownin in instant Pu-erh tea is close to 50% (Supplementary Fig. 8), theabrownin at 225 mg/Kg/day was given (half of the dose of instant Pu-erh tea at 450 mg/Kg/day). The TC and TG levels in serum and liver were decreased with Pu-erh tea and theabrownin intervention. Additionally, the TC and TG levels in both serum and liver from the theabrownin group were significantly lower than those in the Pu-erh tea group (Fig. 5b). Examination of the results of the BA profile revealed that the ileal tauro-conjugated BAs, TCDCA, and TUDCA, were significantly elevated by theabrownin and Pu-erh tea ingestion, but the increase was more remarkable with theabrownin (Fig. 5c). Moreover, theabrownin robustly attenuated ileal BSH-producing microbes and BSH activity in mice with greater effect than Pu-erh tea (Fig. 5d, e).

The ileal microbiota of C57BL/6J mice were cultured in vitro and subsequently treated with Pu-erh tea and theabrownin. BSH activity was found to be dramatically reduced by Pu-erh tea and theabrownin intervention. Furthermore, the BSH activity in the theabrownin treatment group was significantly lower than in the Pu-erh tea treatment group (Fig. 5f). Taken together, these results indicated that theabrownin was directly responsible for the attenuated amounts of BSH-producing bacteria and subsequent decreases in BA hydrolysis, causing higher levels of tauro-conjugated BAs as well as lower levels of serum and hepatic TC, and TG.

In order to investigate whether the treatment with Pu-erh tea/theabrownin depends on the microbiota, germ-free mice after 8 weeks of HFD feeding were transplanted with microbiota from mice fed with HFD or HFD together with theabrownin, respectively. As a result, the mice received microbiota from theabrownin group showed lower weight gain and serum TC and TC concentrations compared to the mice received microbiota from HFD group after 8 weeks of HFD feeding, similar to their donors. (Supplementary Fig. 10). The results showed that the beneficial effects of theabrownin could be transmitted by stool transplantation, indicating that the weight loss and cholesterol-lowering effects of theabrownin were dependent on the gut microbiota.

### Ileal conjugated BAs inhibited FXR-FGF15 to promote BA synthesis

**Fig. 6:**
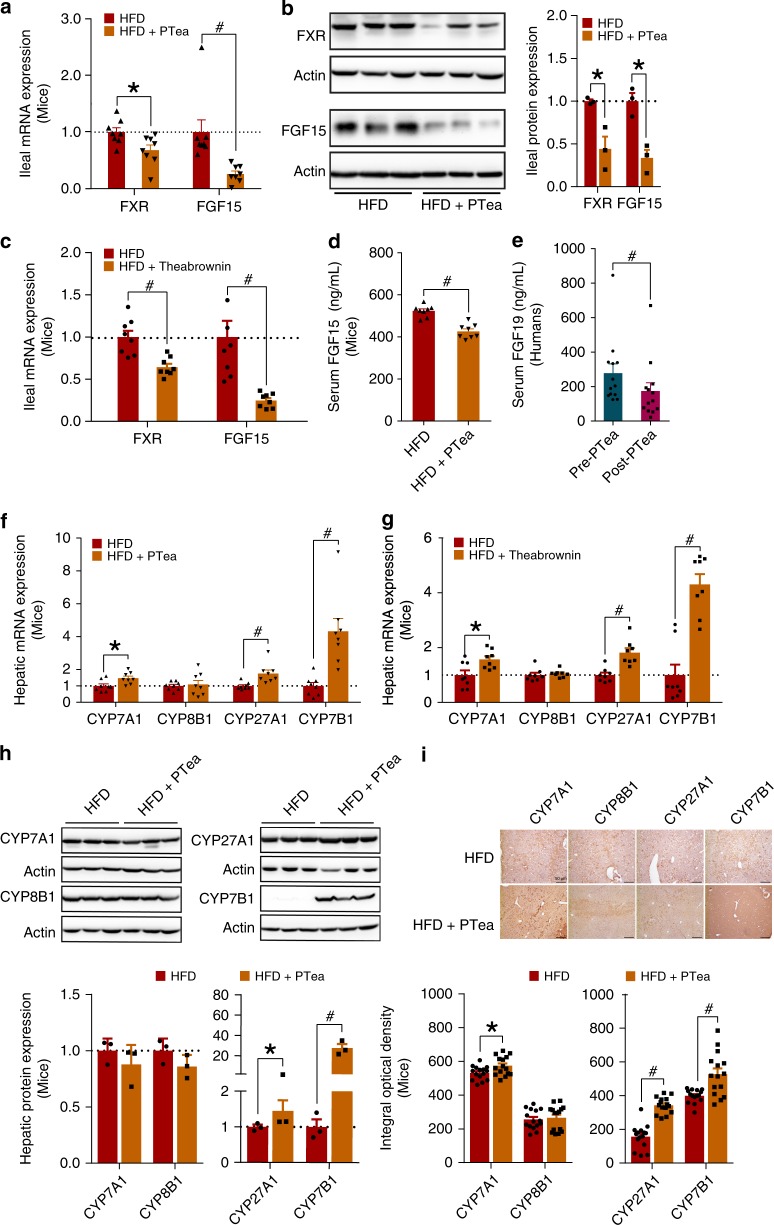
Conjugated BAs inhibited the FXR-FGF15 to induce hepatic BA synthesis. **a** Gene expression of FXR and FGF15 in ileum of mice fed HFD and 450 mg/Kg/day Pu-erh tea for 26 weeks. *n* = 8 individuals/group. **b** FXR and FGF15 protein expression level in ileum of mice fed HFD and 450 mg/Kg/day Pu-erh tea for 26 weeks. *n* = 3 individuals/group. **c** Gene expression of FXR and FGF15 in ileum of mice fed HFD and 225 mg/Kg/day theabrownin for 8 weeks. *n* = 8 individuals/group. **d** Serum FGF15 levels of mice fed HFD and 450 mg/Kg/day Pu-erh tea for 26 weeks. *n* = 8 individuals/group. **e** Serum FGF19 levels of human subjects that received standard diet and 50 mg/Kg/day Pu-erh tea for 4 weeks. *n* = 13 individuals/group. **f** Hepatic mRNA expression levels of BA synthetic enzymes of mice fed HFD and 450 mg/Kg/day Pu-erh tea for 26 weeks. *n* = 8 individuals/group. **g** Hepatic mRNA expression levels of BA synthetic enzymes of mice fed HFD and 225 mg/Kg/day theabrownin for 8 weeks. *n* = 8 individuals/group. **h** Hepatic protein expression levels of BA synthetic enzymes of mice fed HFD and 450 mg/Kg/day Pu-erh tea for 26 weeks. *n* = 3 individuals/group. **i** IHC staining of hepatic BA synthetic proteins of mice fed HFD and 450 mg/Kg/day Pu-erh tea for 26 weeks (scale bar, 50 μm). The mRNA expression and protein expression were normalized to GAPDH and β-Actin, respectively. Data were expressed as mean ± SEM. Differences between data in mice and human were assessed by Mann–Whitney U test and Wilcoxon rank-sum test, respectively, *p < 0.05, ^#^*p* < 0.005

We next investigated the mRNA expression level of FXR and FGF15 in ileum at 26 weeks for Pu-erh tea and at 8 weeks for theabrownin intervention. Both mRNA expression of FGF15 and relative protein levels of FXR and FGF15 in the distal ileum were decreased in both ND + PTea and HFD + PTea groups relative to their controls, and FXR mRNA was reduced in the HFD + PTea group at 26 weeks in the Pu-erh tea experiment (Fig. 6a, b and Supplementary Fig. 11a, b). Similarly, the mRNA expression of FXR and FGF15 were significantly decreased in the theabrownin group compared to the HFD control group (Fig. 6c). In addition, the serum concentration of FGF15 or FGF19 protein, measured by ELISA, was decreased in the Pu-erh treatment groups of mice and human subjects, respectively (Fig. 6d, e and Supplementary Fig. 11c). Both mRNA and protein results suggested that the accumulation of ileal tauro-conjugated BAs may directly down-regulate intestinal FXR-FGF15 signaling.

The hormone, FGF15, when secreted into the portal vein, circulates to its receptor, FGFR4, in the liver and acts to reduce hepatic BA synthesis via suppression of BA synthetic enzymes^11^. Hepatic FGFR4 mRNA expression was found to be decreased in parallel with the decreased ileal FGF15 mRNA expression and serum FGF15 protein levels in mice treated with Pu-erh tea (Supplementary Fig. 11d). The mRNA expression levels for the hepatic BA synthetic genes, CYP7B1 and CYP27A1 were the most significantly increased, CYP7A1 was also elevated but CYP8B1 was not significantly affected in the HFD + PTea group (Fig. 6f and Supplementary Fig. 11e). Similarly, the relative expression of mRNA levels for CYP7B1 and CYP27A1 were significantly increased by theabrownin treatment compared to the HFD control group (Fig. 6g). Moreover, western blotting and IHC histological staining confirmed that Pu-erh tea induced an increase in the protein expression levels of hepatic BA synthetic enzymes, CYP7B1 and CYP27A1 (Fig. 6h, i and Supplementary Fig. 11f, g). Increased conjugation of BAs was correlated with an observed increase in the BA conjugation enzyme, BAAT, which allows conjugation of the primary BAs, CA and CDCA, with glycine or taurine (Supplementary Fig. 12a). Taken together, these results indicated that the decreased ileal FGF15 secretion led to increased BA synthetic enzyme mRNA in the liver, and increased mRNA for genes in the alternative BA synthetic pathway involving CYP7B1 and CYP27A1, which led to increased production of CDCA rather than CA.

As a main metabolic pathway of cholesterol, BA biosynthesis plays a vital role in maintaining the homeostasis of cholesterol levels in liver and serum. The BA pool is comprised of the amount of BAs in the liver, serum, and intestinal tissue. Approximately 5% of total BAs that do not get recycled are then excreted via feces. The loss of BAs is replenished by BA de novo synthesis in liver. Quantitative analysis of 49 BAs in serum, liver, small intestinal contents, cecum contents, colon contents, and feces were done to calculate the BA pool and, in addition, BA excretion measurements were performed in all of the mouse groups at 26 weeks after Pu-erh tea intervention. Pu-erh tea treatment dramatically elevated the BA pool size and fecal BA excretion in both normal chow and HFD mouse groups (Supplementary Figs. 7a and 12b). Concurrently, the total BA loss in human feces was significantly increased after Pu-erh tea consumption (Supplementary Fig. 7b).

Cholesterol in the circulation re-enters the liver by reverse cholesterol transporters for utilization in BA synthesis. The expression of the 3-hydroxy-3-methylglutaryl-CoA-reductase (HMGR) gene, the rate limiting enzyme in cholesterol de novo biosynthesis, was increased and the reverse cholesterol transporters ABCA1 and ABCG1 were highly expressed in the livers of mice on Pu-erh tea intervention (Supplementary Fig. 12c, d). These results indicated that Pu-erh tea consumption elevated hepatic cholesterol biosynthesis and reuptake, increased de novo BA synthesis and increased elimination by fecal loss. The combination of these effects would reasonably be expected to result in a decrease in the hepatic and serum cholesterol level.

### Theabrownin promoted alternative pathway of hepatic BA synthesis

**Fig. 7:**
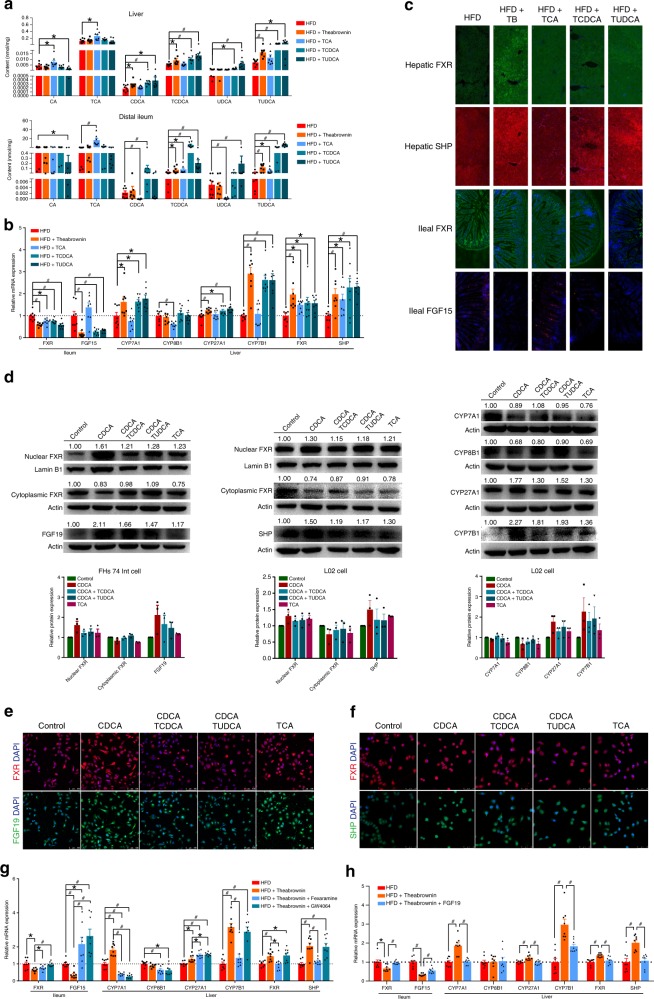
FXR-FGF15 and FXR-SHP regulated BA synthesis in alternative pathway. **a** Dominant BAs in liver and ileum of HFD fed mice gavaged with 225 mg/Kg/day theabrownin and 50 mg/Kg/day TCA, TCDCA, TUDCA respectively for 8 weeks. *n* = 8 individuals/group. **b** mRNA expression of ileal FXR, FGF15 and hepatic FXR, SHP and BA synthetic genes in HFD fed mice gavaged with 225 mg/Kg/day theabrownin and 50 mg/Kg/day TCA, TCDCA, TUDCA respectively for 8 weeks. *n* = 8 individuals/group. **c** Immunofluorescence staining of ileal FXR, FGF15 and hepatic FXR, SHP of HFD fed mice gavaged with 225 mg/Kg/day theabrownin and 50 mg/Kg/day TCA, TCDCA, TUDCA respectively for 8 weeks (scale bar, 50 μm). **d** Fifty micromole per liter of TCDCA and TUDCA inhibited protein expression of nuclear FXR in the human FHs 74 Int and L02 cell lines, and 50 μM of CDCA and TCA activated the expression of nuclear FXR in human FHs 74 Int and L02 cell lines and promoted expression of CYP27A1 and CYP7B1 in alternative pathway of bile acids synthesis. *n* = 3 individuals/group. **e** Immunofluorescence staining of FXR, FGF19 in FHs 74 Int cells supplied with 50 μM CDCA, CDCA coupled with 50 μM of TCDCA, CDCA coupled with 50 μM of TUDCA, and 50 μM of TCA for 24 h (scale bar, 100 μm). **f** Immunofluorescence staining of FXR, SHP in L02 cell lines supplied with 50 μM of CDCA, CDCA coupled with TCDCA, CDCA coupled with TUDCA and TCA for 24 h (scale bar, 50 μm). **g** The mRNA expression of ileal FXR, FGF15 and hepatic BA synthetic genes in HFD fed mice gavaged with 225 mg/Kg/day theabrownin, theabrownin coupled with 100 mg/Kg/day fexaramine for 8 weeks. *n* = 8 individuals/group. **h** The mRNA expression of ileal FXR, FGF15 and hepatic BA synthetic genes in HFD fed mice gavaged with 225 mg/Kg/day theabrownin, theabrownin coupled with 50 μg/Kg/day recombinant FGF19 protein by intraperitoneal injection for 8 weeks. *n* = 8 individuals/group. The mRNA expression was normalized to GAPDH. Protein expression was normalized by β-Actin or Lamin B1. Data were expressed as mean ± SEM. Differences between data were assessed by the Mann–Whitney U test, *p < 0.05, ^#^*p* < 0.005

To determine whether the inhibition of FXR signaling by conjugated BAs was achievable in vivo, HFD mice were orally gavaged with 225 mg/Kg/day of theabrownin and 50 mg/Kg/day of TCA, TCDCA and TUDCA, respectively. Oral administration of theabrownin significantly elevated levels of TCDCA and TUDCA in ileum and liver of mice, while TCA, TCDCA and TUDCA treatment resulted in increased levels of these BAs and their corresponding unconjugated BAs (Fig. 7a). Similar to the theabrownin group, treatment with 50 mg/Kg/day of TCDCA or TUDCA for 8 weeks significantly reduced ileal FXR and FGF15 mRNA expressions and induced the expression of SHP in the liver (Fig. 7b). Increased induction of hepatic CYP7A1, CYP7B1 BA synthetic gene expression occurred in mice treated with TCDCA or TUDCA relative to HFD treated mice, which also resembled the changes induced by theabrownin. The protein expression levels of ileal FXR, FGF15 and hepatic FXR, SHP were further analyzed using immunofluorescence staining. Similar to the mRNA expression results, both TCDCA and TUDCA inhibited FXR and FGF15 protein expression in ileum and elevated FXR-SHP expression levels. Moreover, induction of BA synthesis by theabrownin, TCDCA, or TUDCA for 8 weeks resulted in a significant reduction of hepatic and serum cholesterol levels as well as body weight (Supplementary Fig. 13a). These results indicated that mice treated with TCDCA or TUDCA predominantly inhibited the expression of ileal FXR-FGF15 and induced the expression of hepatic BA synthesis genes involved in the alternative pathway of BA synthesis. Subsequently, elevated CDCA level in the liver activated hepatic FXR-SHP signaling which, in combination with TCDCA/TUDCA, inhibited expression of CYP8B1 in the classical pathway and further induced CYP7B1 expression in the BA alternative pathway.

On the other hand, TCA as a FXR agonist activated ileal FXR-FGF15 and hepatic FXR-SHP signaling which, in turn, inhibited the expression of CYP8B1 in the classical pathway of BA synthesis (Fig. 7b, c). These results suggested a unique mechanism by which theabrownin, through inhibition of intestinal FXR-FGF15, promoted the hepatic FXR-SHP pathway, leading to increased BA synthesis via the alternative pathway.

To validate the inhibition of ileal FXR signaling by conjugated BAs, human FHs 74 Int and L02 cell lines were cultured and treated with several unconjugated and corresponding conjugated BAs including TCDCA and TUDCA. Our data showed that 50 μM of CDCA, as a potent FXR ligand, increased FXR expression in the cell nucleus, which, in ture, up-regulated the expression of FGF19 and SHP in FHs 74 Int and L02 cell lines (Fig. 7d). As a result, CDCA slightly inhibited the expression of CYP7A1 and CYP8B1 in the classical BA synthetic pathway and greatly promoted the expression of CYP27A1 and CYP7B1 in the alternative pathway. An aliquot of 50 μM TCDCA or TUDCA attenuated the ability of CDCA to activate FXR signaling and promote SHP and FGF19 expression (Fig. 7d–f). Therefore, co-treatment with CDCA and TCDCA or TUDCA, which mimics the hepatic BA profile under Pu-erh tea treatment, was able to activate the alternative pathway of BA synthesis to a greater extent, than the classical pathway.

**Fig. 8:**
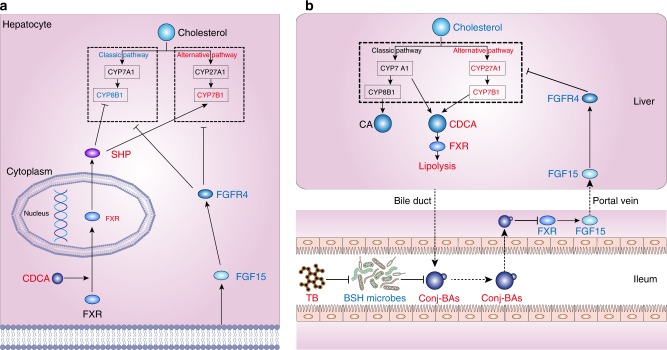
Proposed mechanism for theabrownin reduce cholesterol level. **a** Theabrownin promoted alternative bile acids synthetic pathway by simultaneous inhibition of intestinal FXR-FGF15 signaling with TCDCA or TUDCA and activation of hepatic FXR-SHP signaling with CDCA.Intestinal FXR-FGF15 signaling inhibits hepatic bile acid synthesis genes CYP7A1, CYP8B1, CYP27A1 and CYP7B1 with no selectivity, whereas hepatic FXR-SHP signaling selectively inhibits CYP8B1 in the classic pathway. Theabrownin in Pu-erh tea induced TCDCA and TUDCA in distal ileum to inhibit FGF15 production and thus, activated bile acid synthesis enzymes. Additionally, increased CDCA production in the liver promoted nuclear FXR expression as well as SHP to inhibit CYP8B1 expression. Ultimately, the combined regulation of decreased intestinal FXR-FGF15 and increased hepatic FXR-SHP on bile acid synthesis enzymes resulted in increased expression of CYP7B1 in alternative pathway and decreased expression of CYP8B1 in classic pathway leading to increased production of CDCA rather than CA. **b** Proposed mechanism for theabrownin induced attenuation of hepatic cholesterol levels via modulation of gut microbiome-mediated bile acid metabolism. Primary BAs, mainly CA and CDCA, were produced in the liver from cholesterol and conjugated to glycine or taurine to form conjugated BAs and then secreted to the intestinal. BSH enzymes are produced in intestinal microbes and function to hydrolyze conjugated BAs into unconjugated BAs. BSH microbes were suppressed by theabrownin in Pu-erh tea resulting in the accumulation of conjugated BAs in distal ileum. Conjugated BAs inhibited intestinal FXR-FGF15 signaling which subsequently alleviated the suppression of BA synthesis gene expression by this signaling pathway, resulting in increased BA production in alternative synthetic pathway and fecal BA excretion and ultimately, decreased cholesterol levels

A follow-up recovery study was performed using HFD mice treated with 225 mg/Kg/day of theabrownin, and theabrownin coupled with 100 mg/Kg/day GW4064, an FXR agonist, and fexaramine, a gut-restricted FXR agonist^27,28^ for 8 weeks. The results showed that treatment with GW4064 and fexaramine markedly reversed the theabrownin induced alterations in the expression of ileal FGF15 and hepatic BA synthetic genes CYP7A1 (Fig. 7g). Interestingly, the hepatic SHP expression levels were significantly elevated by GW4064 rather than the gut-selective FXR agonist fexaramine, which acted together with increased FGF15 to inhibit CYP7A1 and CYP8B1 whereas the CYP7B1 was not affected compared to theabrownin group (Fig. 7g). More importantly, selective activation of intestinal FXR-FGF15 by fexaramine could reverse the increased expression levels of hepatic SHP and CYP7B1 and decreased body weight, TC and TG levels induced by theabrownin (Fig. 7g and Supplementary Fig. 13b). We further treated HFD fed mice with theabrownin, Z-Guggulsterone, a gut-specific FXR antagonist, and the combination of theabrownin and Z-Guggulsterone for 8 weeks to investigate the role of intestinal FXR in the TC/TG-lowering effects of theabrownin. The results revealed that the body weight and TC, TG concentration in serum were decreased in theabrownin and Z-Guggulsterone groups compared to HFD mice which confirmed the cholesterol-lowering effect of Pu-erh tea was dependent on the attenuation of intestinal FXR signaling. When FXR signaling was attenuated by Z-Guggulsterone, theabrownin treatment could not further reduce the serum TC and TG level. The results revealed that selectively attenuated intestinal FXR could reduce TC and TG level as theabrownin does. The effects of theabrownin on serum TC and TG was dependent on the inhibition of intestinal FXR signaling (Supplementary Fig. 13c). These results confirmed that the promotion of the alternative BA synthetic pathway by theabrownin depends on the simultaneous inhibition of intestinal FXR-FGF15 signaling and activation of hepatic FXR-SHP signaling (Fig. 8a).

Examination of the effect of recombinant FGF19 protein in vitro directly on BA synthetic enzymes and cholesterol levels was performed on the normal human hepatocyte L02 cell line that was cultured and treated with different concentrations (25, 50, 75, 100 ng/mL) of recombinant FGF19 protein. The BA synthetic protein levels were analyzed by Western Blot and the protein expression levels of BA synthetic enzymes were gradually reduced as the concentration of added FGF19 protein was increased (Supplementary Fig. 13e).

Further, HFD mice were treated with 225 mg/Kg/day theabrownin or theabrownin coupled with 50 μg/Kg/day of recombinant FGF19 protein to verify whether FGF19 was a key factor in the cholesterol-lowering mechanism of theabrownin in vivo. The effects of theabrownin on mRNA expression levels of hepatic BA synthetic enzymes were reversed by recombinant FGF19 injection (Fig. 7h). Recombinant FGF19 protein treatment induced an increase of cholesterol levels and body weights relative to the theabrownin treated group (Supplementary Fig. 13d). FGF19 protein supplementation directly suppressed BA synthetic gene expression in vitro leading to increased TC levels in vivo (Supplementary Fig. 13d).

To confirm the roles of CYP7A1 and CYP7B1 in the cholesterol-lowering effects of theabrownin, we used hepatic AAV-shRNA to knockdown CYP7A1 and CYP7B1, respectively, in the mice and then followed with 8-weeks of theabrownin treatment. We found that the hepatic AAV-shRNA knockdown of CYP7B1 eliminated the cholesterol-lowering effects of Pu-erh tea and theabrownin, while theabrownin still could lower the cholesterol level in hepatic AAV-shRNA knockdown of CYP7A1. These results indicated that the major cholesterol-lowering effects of Pu-erh tea and theabrownin is due to elevated CYP7B1 expression (Supplementary Fig. 14).

## Discussion

The lipid and cholesterol-lowering effects of Pu-erh tea have been shown to be of clinical value in treating obesity, fatty liver and NAFLD. Specifically, Pu-erh tea was found to lower TG and TC levels more significantly than green, oolong, or black teas^3^. In this study, mice and human subjects receiving Pu-erh tea exhibited significant decreases in hepatic and serum cholesterol levels. These results confirmed other reported lipid and cholesterol-lowering effects of Pu-erh tea in both mice and human subjects^29–31^.

BSH catalyzes the “gateway” reaction—the hydrolysis of the amino group from the conjugated BAs to produce unconjugated BAs, which is a prerequisite for the subsequent 7α/β-dehydroxylation reactions producing secondary BAs^32,33^. A previous study showed that the antioxidant, tempol, preferentially reduced the relative abundance of the *Lactobacillus* genus, decreased BSH activity, and led to changes in BA composition that ultimately improved obesity^15^. Inhibition of BA deconjugating microbes by administration of ampicillin decreased the expression of ileal FXR target genes and increased the levels of primary TCA and T-βMCA^34^. As reported, the BSH activity was mainly attributed to Lactobacilli and Bifidobacteria^35–37^. Our experimental analyses of the gut microbiota showed that there were decreased relative abundances of BSH-producing *Lactobacillus, Bacillus, Streptococcus and Lactococcus* genera in humans and mice with Pu-erh/theabrownin treatment as well as decreased BSH activity in the ileal lumen of mice. Additionally, when theabrownin was administered to HFD fed mice, intestinal BSH activity and serum and liver cholesterol levels were significantly reduced. These results revealed that Pu-erh tea derived theabrownin directly inhibited the activity of intestinal BSH enzymes, suppressed BA deconjugation in the small intestine, and impacted hepatic and serum cholesterol levels.

BAs are also endogenous signaling molecules that bind to the BA receptors, FXR and TGR5, to regulate BA homeostasis in the enterohepatic circulation, modulate cholesterol and triglyceride metabolism, and maintain glucose and energy homeostasis^38,39^. Potent endogenous ligands of FXR include mainly unconjugated BAs such as CA, CDCA, DCA, LCA, and UDCA^7,40^. The gut microbiota and BA metabolism are closely interrelated and this relationship is directly modulated by the intestinal-hepatic FXR-FGF15-FGFR4 signaling axis. T-βMCA and T-αMCA were identified as naturally occurring FXR antagonists and reduced levels of T-βMCA led to a decreased production of FGF15^15^. Ampicillin-induced decrease of ileal FXR signaling was recovered by oral administration of unconjugated CA but not conjugated TCA, indicating the importance of BA deconjugation for FXR activation^34^. In our experiments, a decreased level of intestinal BSH activity resulted in an elevation of the tauro-conjugated BAs including TCDCA and TUDCA in the distal ileum. UDCA has long been regarded as a secondary BA epimerized by microbiota in mice^41^. However, there was a recent study that supports the notion that UDCA is a primary BA in mice^15^, as significant levels of UDCA were identified in germ-free mice. We hypothesized that in addition to T-βMCA which only exists in rodents, TCDCA and TUDCA which exist in both rodents and humans with relatively high concentrations in the intestinal lumen, are antagonistic to ileal FXR, similar to T-βMCA. In order to validate this hypothesis, a cell study was conducted on human L02 and FHs 74 Int cell lines which were treated with CDCA, TCA, CDCA coupled with TCDCA, and CDCA coupled with TUDCA, respectively. As a result, FXR was activated by CDCA and TCA moving from cytoplasm to nucleus, and subsequently activating the expression of FGF15 or SHP. On the contrary, TCDCA and TUDCA reversed the activation of FXR signaling by CDCA via a reduction of the portion of FXR in nucleus. A validation experiment was conducted in vivo by treating HFD fed mice with a conjugated BA (TCDCA or TUDCA) which ultimately resulted in reduced expression of ileal FXR-FGF15 signaling and elevated BA synthesis. These results provided supporting evidance that the increased level of ileal conjugated BAs during Pu-erh tea treatment inhibited FXR transcription of FGF15 in the intestine and promoted hepatic BA synthesis. Moreover, Z-Guggulsterone, a gut-selective FXR antagonist, could reduce the cholesterol level and body weight of mice similar to results seen using theabrownin. Meanwhile, antagonizing intestinal FXR eliminated the cholesterol-lowering effects of theabrownin, suggesting that inhibition of intestinal FXR is the key step towards theabrownin-induced cholesterol-lowering outcome. Additionally, using a non-selective FXR agonist, GW4064, did not significantly affect the theabrownin induced alternative pathway in bile acids synthesis. Administration of the gut-restricted FXR agonist fexaramine or supplementation with recombinant FGF19 protein abolished the theabrownin-induced reduction in FGF15 along with increased BA synthetic enzymes in alternative pathway. Taken together, these results provided strong evidence that inhibition of the FXR signaling pathway in the intestine together with activation of the FXR signaling pathway and alternative BA synthetic pathway in the liver could be a viable therapy for treating HFD-induced obesity and hypercholesterolemia.

Other previous research has revealed that gut microbiota regulated expression of FGF15 in the ileum and CYP7A1 levels in the liver by an FXR-dependent mechanism^42^. It has also been reported that enhanced fecal BA loss is accompanied by enhanced hepatic BA synthesis^43,44^. These findings were further confirmed in our study, in that inhibition of intestinal FXR resulted in an enhancement of hepatic BA synthetic gene mRNA expression along with an increased BA pool and increased fecal BA loss. A unique metabolic feature identified in this study was that Pu-erh tea-derived theabrownin stimulated enzyme activity in the alternative pathway for BA synthesis, ie, a shift from CA to CDCA production and accumulation of non-12α-hydroxylated BAs. Promotion of BA production ultimately leads to the increased consumption of cholesterol in the liver.

According to the reports^45^, transgenic mice expressing an FGF19 analog in the muscle have decreased serum cholesterol. This is inconsistent with our hypothesis that decreasing FGF15 increases BA synthesis and decreases cholesterol level. Muscle FGF19 may predominantly manipulate energy expenditure and fat mass. In our research, expression of FGF15 or intraperitoneal injection of FGF19 predominantly recycled into the liver via the portal vain, and further combined with the receptor of FGFR4 to attenuate the expression of BA synthesis genes. We, therefore, proposed that the cholesterol-lowering effect of Pu-erh tea is achieved by a combination of decreased ileal FXR-FGF15 and increased hepatic FXR-SHP signaling.

In summary, the mechanism by which the Pu-erh tea derived theabrownin reduces cholesterol levels in liver and plasma involves the following (Fig. 8b): (1) decreased relative abundance of BSH-producing microbes induced by the theabrownin in Pu-erh tea, (2) increased conjugated BAs, especially TCDCA and TUDCA that act in an antagonistic manner on intestinal FXR, (3) decreased activation of intestinal FXR resulting in decreased production of FGF15/FGF19 along with subsequent reduced FGF15/FGF19-FGFR4 signaling coupled with increased activation of hepatic FXR-SHP signaling in the liver, (4) increased de novo BA synthesis in the alternative pathway and reverse transport of cholesterol into liver, and (5) increased elimination of BAs via the feces.

Further studies on human subjects are warranted to better assess the effects of long-term use of Pu-erh tea or theabrownin on managing weight loss and improving hypercholesterolemia. Female subjects should also be used to take into account the gender factor. In addition, evaluation of theabrownin side effects and optimal dosage for humans will require clinical trials before it can become a routine therapy. These preliminary studies, however, do show promise for the efficacy of theabrownin in the treatment of hypercholesterolemia, obesity, and other metabolic disorders such as NAFLD.

## Methods

### Chemicals and reagents

Control diet contained 10% lipids, 19% proteins, and 71% carbohydrates, while the high fat diet contained 45% lipids, 19% proteins, and 36% carbohydrates. The Pu-erh tea infusions were prepared by dissolving 600 mg of tea powder (scented Deepure, Tasly holding group, Tianjin, China) with 200 mL pure sterilized water. The antibodies used for western blot were: FXR (Abcam, ab28480), FXR (Biorbyt, orb156973), FGF15 (Santa Cruz, sc398338), FGF19 (Abcam, ab85042), SHP (Abcam, ab186874), CYP7A1 (Abcam, ab65596), CYP8B1 (Abcam, ab191910), CYP27A1 (Abcam, ab126785), CYP7B1 (Abcam, ab138497), β-Actin (CST, 4970S), Lamin B1 (Beyotime, AF1408), anti-mouse IgG (CST, 7076S), anti-rabbit IgG (CST, 7074S). All BA standards were obtained from Sigma-Aldrich and Steraloids. The mouse FGF15 ELISA kit was purchased from LifeSpanBioScience, Inc (LS-F11446). The human FGF19 ELISA kit was purchased from Antibody and Immunoassy Services (31200). The reagents used in the animal study included TCA (J&K Scientific, 909688), TCDCA (Matrix Scientific, 100646), TUDCA (J&K Scientific, 496672), Z-Guggulsterone (BioChemPartner, BCP07472), Fexaramine (BioChemPartner, BCP15784) and Recombinant human FGF19 protein (R&D system, 969-FG/CF).

### Animal study

All animal procedures and testing were performed according to the national legislation and local guidelines of the Laboratory Animals Center at Shanghai Jiao Tong University, Shanghai, China. 3-week-old C57BL/6J male mice were purchased from Shanghai Laboratory Animal Co. Ltd. (SLAC, Shanghai, China). All the mice were maintained in a specific-pathogen-free (SPF) environment with controlled conditions, a 12 h light/dark cycle at 20–22 °C and 45 ± 5% humidity. The Pu-erh tea infusions were prepared by dissolving 600 mg of tea powder (scented Deepure, Tasly holding group, Tianjin, China) with 200 mL pure sterilized water.

In the Pu-erh tea intervention study, 3-week-old mice were acclimated by placing them on a control chow diet administered ad libitum for one week and then they were randomly divided into four groups, eight mice per group: control group (ND) that received normal chow diet and autoclaved, sterilized water, Pu-erh tea infusion group (ND + PTea) that received chow diet with 3 mg/mL of Pu-erh tea, a high fat diet group (HFD) and high fat diet with 3 mg/mL Pu-erh tea infusion group (HFD + PTea). The dosage of Pu-erh tea was 450 mg/Kg per day. All the mice were raised with free access to control chow/HFD and water/tea infusion, and their body weights, food intakes and tea/water consumptions were recorded once a week for 26 weeks.

In the preventive treatment study, mice fed HFD for 4, 22, and 42 weeks were supplied with 3 mg/mL instant Pu-erh tea infusion (450 mg/Kg/day) for 4 weeks respectively (HFD4 + PTea, HFD22 + PTea, HFD42 + PTea groups) and the control groups were fed HFD for 8, 26, 46 weeks, respectively (HFD8, HFD26, HFD46 groups), eight mice per group.

In the theabrownin study, HFD fed mice were supplied with 3 mg/mL instant Pu-erh tea infusion (450 mg/Kg/day, HFD + PTea group) or 1.5 mg/mL theabrownin infusion (225 mg/Kg/day, HFD + Theabrownin group) with free food access for 8 weeks, eight mice per group.

In the second theabrownin intervention study, 3-week-old mice were acclimated by placing them on a control chow diet ad libitum for one week and then randomly dividing them into four groups, eight mice per group: (1) control group (ND) received normal chow diet and autoclaved, sterilized water, (2) chow diet with 1.5 mg/mL theabrownin infusion group (ND + Theabrownin), (3) high fat diet group (HFD), and (4) HFD with 1.5 mg/mL theabrownin infusion group (HFD + Theabrownin). The dosage of theabrownin was 225 mg/Kg per day and the intervention lasted for 8 weeks.

In the fecal microbiota transplantation study, the microbiota donors were mice treated with HFD or HFD and theabrownin for 8 weeks. Feces of the donors were collected and dispersed in sterile Ringer working buffer, the supernatant were mixed with skimmed milk for transplantation. Four-week-old germ-free male C57BL/6J mice were randomly divided into two groups (7 each group), housed in sterile plastic package isolators (each group for one isolator) and supplied with sterilized normal diet. After a 2-week acclimation, germ-free mice were oral gavaged with fecal suspension from mice with HFD or HFD and theabrownin. The oral gavage was repeated in the next two days to reinforce the microbiota transplantation. The transplanted mice were then supplied with HFD for another 8 weeks. The body weight was recorded once a week and blood samples were collected at the end of the experiment for analysis of TC and TG.

In the BA treatment study, 3-week-old mice were adapted with HFD for one week and subsequently divided into five groups, eight mice for each group: (1) vehicle fed HFD (HFD group), (2) HFD supplied with 1.5 mg/mL theabrownin infusion (HFD + Theabrownin group), (3) HFD coupled with 50 mg/Kg body weight of TCA by gavage (HFD + TCA group), (4) HFD coupled with 50 mg/Kg body weight of TCDCA by gavage (HFD + TCDCA group), and (5) HFD coupled with 50 mg/Kg body weight of TUDCA by gavage (HFD + TUDCA group). The interventions were conducted for 8 weeks.

For the FXR regulation study, 3-week-old HFD fed mice were acclimated for one week, divided into three groups, eight mice per group, (1) vehicle fed HFD (HFD group), (2) HFD supplied with 1.5 mg/mL theabrownin infusion with free access (HFD + Theabrownin group) and finally, (3) supplied with 1.5 mg/mL theabrownin with free access coupled with 100 mg/Kg Fexaramine per day by gavage (HFD + Theabrownin + Fexaramine group) or 100 mg/Kg GW4064 per day by gavage (HFD + Theabrownin + GW4064 group).

For the second FXR regulation study, 3-week-old HFD fed mice were acclimated for one week, divided into four groups, eight each group, (1) vehicle fed HFD (HFD group), (2) HFD supplied with 1.5 mg/mL theabrownin infusion with free access (HFD + Theabrownin group), (3) HFD supplied with 100 mg/Kg/day Z-Guggulsterone by gavage (HFD + Z-Guggulsterone group), and (4) HFD supplied with 1.5 mg/mL theabrownin with free food access coupled with 100 mg/Kg Z-Guggulsterone per day by gavage (HFD + Theabrownin + Z-Guggulsterone group).

For the FGF19 intervention study, 3-week-old HFD fed mice were acclimated for 1 week, divided into three groups, eight mice per group, (1) vehicle fed HFD (HFD group), (2) HFD supplied with 1.5 mg/mL theabrownin infusion with free food access (HFD + Theabrownin group) and, (3) HFD supplied with 1.5 mg/mL theabrownin with free food access coupled with 50 μg/Kg recombinant FGF19 protein per day by intraperitioneal injection (HFD + Theabrownin + FGF19 group). The interventions were sustained for 8 weeks.

In the CYP7A1 and CYP7B1 study, 4-week-old mice fed HFD were acclimated for one week, divided into three groups, 16 each group, (1) vehicle fed HFD (shControl), (2) HFD with intraperitioneal injection of AAV-shRNA of CYP7A1 (shCYP7A1), and (3) HFD with intraperitioneal injection of AAV-shRNA of CYP7B1 (shCYP7B1) and maintained for 3 weeks to knockdown CYP7A1 and CYP7B1. Afterwards, all the mice were maintained with HFD and half in each group were supplied with 1.5 mg/mL theabrownin infusion (225 mg/Kg/day) for another 8 weeks. Blood samples were collected at the end of the experiment for analysis of TC concentrations.

At the end of these experiments, mice were fasted overnight before being euthanized. Blood samples were collected and then kept at room temperature for half an hour to ensure complete clotting before centrifugation at 4 °C, 5000 rpm for 10 minutes to obtain the serum sample. Tissues including liver, intestinal contents and intestinal tissues and feces were carefully collected and kept in liquid nitrogen and then stored at −80 °C until analysis.

### Distinct components among green tea, black tea, and Pu-erh tea

Comparative studies using rodents treated with Pu-erh tea, green tea, and black tea suggested that fully fermented Pu-erh tea had more effective anti-hyperlipidemic and anti-hypercholesterolemic effects compared to other partially fermented and non-fermented teas. Therefore, certain components generated in the unique fermentation process of Pu-erh tea may possess stronger activity than those in the other teas. The differences in active compounds in green tea, black tea and Pu-erh tea have also been widely investigated. Green tea was found to be enriched in polyphenols, such as catechin and its derivatives, catechingallate (CG), epigallocatechin (EGC), epigallocatechingallate (EGCG), epicatechin (EC), epicatechingallate (ECG), and gallocatechingallate (GCG). Catechin and its derivatives were significantly reduced in Pu-erh tea, presumably due to their longer fermentation processes. Regarding specific manufacturing processes of different teas, the processing of green tea has been designed to avoid the oxidation of polyphenols via oxidase enzymes whereas, the processing of black tea and Pu-erh tea has been designed to promote the oxidation of polyphenols. During the fermentation process, the catechins and their gallate derivatives are oxidized to complex phenolic tea pigments including theaflavins (TF), thearubigins (TR) and theabrownins (TB). TF undergo further oxidation to form the more polymerized TR which is then condensed to TB. To summarize, catechins, TF and TR are reduced in concentration while TB is greatly increased during the Pu-erh tea fermentation process, indicating that TB is the characteristic constituent of Pu-erh tea and may be the bioactive substance that leads to the anti-hypercholesterolemic and anti-hyperlipidemic effects of Pu-erh tea.

### Extraction of instant Pu-erh tea

Instant Pu-erh tea is produced using a specific, standardized manufacturer’s protocol. Briefly, the ripe Pu-erh tea was extracted with water using a multi-stage countercurrent extraction (MCEE) method and spray dried to obtain the instant Pu-erh tea powder for use.

### Theabrownin extraction

Three hundred fifty grams of Pu-erh tea were milled into powder, suspended in a 10 fold volume of absolute ethanol, mixed for 12 h and filtered under vacuum. The residue was extracted with a 10-fold volume of boiled distilled water, kept at 83 °C for 20 min with continuous stirring and then filtered under vacuum. The extraction process was repeated three times, the extracts were combined and then vacuum evaporated to one fifth of the total volume. The concentrated solution was then subjected to a series of liquid-liquid extraction processes, including equal volumes of chloroform, ethyl acetate and n-butanol for 2, 3, 4 times, respectively. The water layers were evaporated to one quarter of their total volume and absolute ethanol added to a final proportion of 85% to precipitate the theabrownin crude extracts.

The extracted crude theabrownin was further purified using a Sevage method. In general, the theabrownin samples were dissolved in distilled water and extracted with a chloroform/n-butanol mixture (5:1, v/v) repeatedly until no precipitated white turbidity was present at the liquid junction region. Absolute ethanol to a proportion of 85% was added to the water layer to precipitate the deproteinized theabrownin. The purified theabrownin was filtered and lyophilized for use.

### Metabolomic study of human subjects

This study was conducted in accordance with the established ethical guidelines and approved by the research ethics committee of the School of Pharmacy, Shanghai Jiao Tong University, Shanghai, China. Written informed consent was obtained from all the subjects before study initiation.

Thirteen healthy male volunteers were enrolled in this study. The age of the participants ranged from 24 to 32 years. with BMIs that ranged from 21.6 to 26.1. The instant Pu-erh tea was prepared by dissolving 1 g of powder in 200 mL of boiling water. All volunteers were provided with standard meals three times a day for a week before tea intervention and subsequently received 300 mL of tea infusion at 8:00 and 20:00 after standard meals for 4 weeks at a dose of 50 mg/Kg/day. No other diets or drinks were consumed during the experimental period. Serum and fecal samples were collected at the end of the first adjustment week (Pre-Tea) and the fourth tea intervention week (Post-Tea) before the first meal. Samples were stored at −80 °C until analysis.

### BA analysis

The BA concentration in samples were quantified using ultra performance liquid chromatography coupled with triple quadrupole mass spectrometry (UPLC-TQMS, Waters, Milford, MA) according to a protocol we previously established^20,46^.

### Measurement of serum biochemical indicators and hepatic TC, TG

The serum TC and TG were measured using a TBA-40FR Fully Automatic Biochemical Analyzer (TOSHIBA, Japan), according to manufacturer’s protocol. Hepatic lipids were extracted by the Folch method, briefly, the liver tissues were homogenized with a choroform/methanol (2/1, v/v) solution to a final volume 20 times that of the tissue sample and followed by a series of dispersion, agitation, centrifugation steps. The hepatic levels of total cholesterol (TC) and triglyceride (TG) were measured using Elisa kits (BluGene Biotech, Shanghai, China) according to the manufacturer’s instructions.

### 16S rRNA gene sequencing

Total bacterial genomic DNA samples were extracted using the Fast DNA SPIN extraction kits (MP Biomedicals, Santa Ana, CA, USA), following the manufacturer’s instructions. The quantity and quality of extracted DNA were measured using a NanoDrop ND-1000 spectrophotometer (Thermo Fisher Scientific, Waltham, MA,USA) and agarose gel electrophoresis, respectively.

PCR amplification of the bacterial 16S rRNA gene V4–V5 region was performed using the forward primer (5′-GTGCCAGCMGCCGCGGTAA-3′) and the reverse primer (5′-CCGTCAATTCMTTTRAGTTT-3′). Sample-specific seven-bp barcodes were incorporated into the primers for multiplex sequencing. The PCR components contained 5 μl of Q5 reaction buffer (5×), 5 μl of Q5 High-Fidelity GC buffer (5×), 0.25 μl of Q5 High-Fidelity DNA Polymerase (5U/μl), 2 μl (2.5 mM) of dNTPs, 1 μl (10 uM) of each Forward and Reverse primer, 2 μl of DNA Template, and 8.75 μl of ddH_2_O. Thermal cycling consisted of initial denaturation at 98 °C for 2 min, followed by 25 cycles consisting of denaturation at 98 °C for 15 s, annealing at 55 °C for 30 s, and extension at 72 °C for 30 s, with a final extension of 5 min at 72 °C.PCR amplicons were purified using Agencourt AMPure Beads (Beckman Coulter, Indianapolis, IN) and quantified using the PicoGreen dsDNA Assay Kit (Invitrogen, Carlsbad, CA, USA). After the individual quantification step, amplicons were pooled in equal amounts, and pair-end 2 × 300 bp sequencing was performed using the IllluminaMiSeq platform with MiSeq Reagent Kit v3 at Shanghai Personal Biotechnology Co., Ltd (Shanghai, China).

The Quantitative Insights into Microbial Ecology (QIIME, v1.8.0) pipeline was employed to process the sequencing data. Briefly, raw sequencing reads with exact matches to the barcodes were assigned to respective samples and identified as valid sequences. The low-quality sequences were filtered using the following criteria: sequences that had a length of <150 bp, sequences that had average Phred scores of <20, sequences that contained ambiguous bases and sequences that contained mononucleotide repeats of >8 bp. Paired-end reads were assembled using FLASH. After chimera detection, the remaining high-quality sequences were clustered into operational taxonomic units (OTUs) at 97% sequence identity by UCLUST. A representative sequence was selected from each OTU using default parameters.OTU taxonomic classification was conducted by BLAST searching the representative sequences set against the Greengenes Database using the best hit. An OTU table was further generated to record the abundance of each OTU in each sample and the taxonomy of these OTUs. OTUs containing less than 0.001% of total sequences across all samples were discarded. To minimize the difference of sequencing depth across samples, an averaged, rounded rarefied OTU table was generated by averaging 100 evenly re-sampled OTU subsets under the 90% of the minimum sequencing depth for further analysis at Shanghai Personal Biotechnology Co., Ltd (Shanghai, China).

### Metagenomic analysis

Total microbial genomic DNA samples were extracted using the DNeasyPowerSoil Kit (QIAGEN, Inc., Netherlands), following the manufacturer’s instructions. The quantity and quality of extracted DNAs were measured using a NanoDrop ND-1000 spectrophotometer (Thermo Fisher Scientific, Waltham, MA,USA) and agarose gel electrophoresis, respectively. The extracted microbial DNA was processed to construct metagenome shotgun sequencing libraries with insert sizes of 400 bp by using the Illumina TruSeq Nano DNA LT Library Preparation Kit. Each library was sequenced by the Illumina HiSeq X-ten platform (Illumina, USA) with PE150 strategy at Personal Biotechnology Co., Ltd. (Shanghai, China).

Raw sequencing reads were processed to obtain quality-filtered reads for further analysis. The sequencing adapters were removed from sequencing reads using Cutadapt (v1.2.1). Low quality reads were trimmed by using a sliding-window algorithm. The sequencing reads were aligned to the host genome using BWA to remove host contamination. Once quality-filtered reads were obtained, they were de novo assembled to construct the metagenome for each sample by IDBA-UD (Iterative De Bruijn graph Assembler for sequencing data with highly Uneven Depth). All coding regions (CDS) of metagenomic scaffolds longer than 300 bp were predicted by MetaGeneMark. CDS sequences of all samples were clustered by CD-HIT at 90% protein sequence identity, to obtain a non-redundant gene catalog. Gene abundance in each sample was estimated by soap.coverage based on the number of aligned reads. The lowest common ancestor taxonomy of the non-redundant genes was obtained by aligning them against the NCBI-NT database by BLASTN (*e* value < 0.001). Similarly, the functional profiles of the non-redundant genes were obtained by annotation against the KEGG and EggNOG databases using the DIAMOND alignment algorithm at Shanghai Personal Biotechnology Co., Ltd (Shanghai, China).

### Fecal microbiota transplantation

In the fecal microbiota transplantation study, the microbiota donors were mice treated with HFD or HFD and theabrownin (eight mice each group) for 8 weeks. Feces of the donors were collected at the end of week 8, and a pooled sample in each group was used in the following experiment. An aliquot of 500 mg of pooled sample was dispersed in 25 mL of sterile Ringer working buffer in an anaerobic incubator that was continuously supplied with a gas mixture consisting of N2/H2/CO2 (85:10:5). The solution was suspended by vortexing for 10 min, then precipitated for 10 min. The supernatant was transferred to new sterile tubes and an equal volume of skimmed milk (20%) was added and mixed for transplantation. Four week-old germ-free male C57BL/6J mice were randomly divided into two groups (7 each group), housed in sterile plastic package isolators (each group for one isolator) and supplied with sterilized normal diet. After a 2-week acclimation, germ-free mice were oral gavaged with 150 μL of fecal suspension from mice with HFD or HFD and theabrownin. The same manipulations were repeated in the next two days to reinforce the microbiota transplantation. The transplanted mice were then supplied with HFD for another 8 weeks. Blood samples were collected at the end of the experiment for analysis of TC and TG.

### BSH analysis

Ileum content and fecal samples (50 mg) were dispersed in 250 μL of PBS (pH7.4) and homogenized by vortexing for 1 min. The bacterial cells were lysed using sonication for 90 seconds with a 30 seconds interval in an ice bath. The lysates were then centrifuged at 4 °C, 15,000 rpm for 30 min and the supernatants were transferred to new tubes. The protein solutions were partially diluted with 10 fold volumes of PBS to determine the protein concentration using a BCA Protein Assay Kit (Pierce, Rockford, IL, USA) against a BSA standard according to the manufacturer’s instructions. The original protein solutions were diluted to 2 mg/mL by PBS as the protein working solution. The BSH activities were predicted by generation of d4-CDCA from d4-TCDCA by BSH proteins. The incubation was carried out in 200 uL of 3 mM sodium acetate buffer (pH5.2) which contained 0.1 mM d4-TCDCA and 0.1 mg/mL protein. The mixtures were incubated for 20 min at 37 °C and the reactions were stopped by plunging the samples into dry ice. 100 µL of methanol were added to the mixture, samples were vortexed for 5 min and then centrifuged for 20 min at 4 °C, 15,000 rpm. The supernatants were transferred to sampling vials for d4-CDCA quantification by UPLC-TQMS (Waters, Milford, MA, USA) to determine the BSH activity.

### Preparation and culture of ileal contents

Eight week old C57BL/6J male mice were euthanized. The distal ileum (lower third of the small intestine) was isolated and the fats were stripped away from the gut lumen in a sterile bio-safety cabinet. The ileum tissues were directly transferred to a sterile anaerobic incubator (YQX-II, Shanghai, China) and blood was cleared away using alcohol wipes. Both ends of the ileum tissue were clipped off and discarded. The rest of the tissues were cut longitudinally. Then the intestinal contents were transferred into sterile EP tubes. Five mL of 1 M HEPES buffer solution (Gibco, Life Technologies) were pipetted into 1 g of intestinal contents and subsequently dispersed with gentle agitation to make the bacteria stock solution. All these procedures were conducted in an ice bath and anaerobic incubator that was continuously supplied with a gas mixture consisting of N_2_/H_2_/CO_2_ (85:10:5). A 90 μL aliquot of the bacteria solution was pipetted into a 1.5 mL sterile EP tube and then 10 μL of PBS were added. Pu-erh tea infusion (3 mg/mL) and theabrownin solution (1.5 mg/mL) were then added. The tubes were placed in a 2.5 L anaerobic cultivation pot (MGC, Japan) with a microaerobicaerogenesis pack (MGC, Japan) and an oxygen indicator pack (MGC, Japan) and subsequently cultivated using a shaking incubator set at 37 °C and 150 rpm for 24 h. The cultivation was terminated by directly plunging the tubes into dry ice. The BSH activities of the samples were measured using the method described in the BSH analysis section above.

### Serum FGF15 and FGF19 detection

The serum FGF15 levels of mice were detected using a sandwich ELISA Kit (LifeSpanBioSciences, Inc., Seattle, WA) following the manufacturer’s instructions. Briefly, 100 μL of standards, blank or samples were added to wells which had been pre-coated with the target specific capture antibody of FGF15, which will bind to the target antigen (FGF15). Then 100 μL of biotin-conjugated detection antibody (Detection Reagent A) was added to each well which, in turn, binds to the capture antigen, and the total mixture was incubated for 1 h at 37 °C followed by a wash step with 350 μL wash buffer per well x3.A100 μL aliquot of avidin-horseradish peroxidase (HRP) conjugate (Detection Reagent B) was then added (binds to the biotin) and the plate was incubated for 1 hour at 37 °C and further washed 5 times. About 90 μL of TMB substrate was added to each well followed by incubation for 30 min at 37 °C to allow reaction with the HRP enzyme for detection. Subsequently, 50 μL of sulfuric acid stop solution were added to each well to terminate the color development reaction. The optical density (OD) of each well was measured at a wavelength of 450 nm using a SpextraMax i3 Multi-mode Microplate Reader (Molecular Devices, USA). The standard stock solution was diluted with sample diluent to prepare a standard dilution series from 78 to 5000 pg/mL in order to generate a standard curve. A four parameter logistic curve fit was selected to generate the standard curve and calculate the concentration of each sample.

The serum FGF19 levels of human samples were quantified using a sandwich ELISA Kit (AIS, HongKong, China) according to the manufacturer’s instructions. 100 μL of standards and serum samples were pipetted to wells which were pre-coated with a rabbit polyclonal antibody specific for human FGF-19 to bind human FGF-19 followed by incubation for 1 h at room temperature. After washing away unbounded substances, a 100 μL aliquot of biotin labeled polyclonal detection antibody specific for human FGF19 was added to the wells followed by incubation for 1 h at room temperature. The wells were washed and 100 μL of astreptavidin-HRP conjugate solution were pipetted to each well followed by incubation for 20 min at room temperature. Then 100 μL of HRP substrate solution were added to each well and color development occurred after 15 min of incubation at room temperature. The color development was stopped by adding stop solution and the optical density (OD) of the wells was measured using a SpextraMax i3 Multi-mode Microplate Reader (Molecular Devices, USA). The standard stock solution was diluted with sample diluent and a standard dilution series from 31.2 to 2,000 pg/mL was prepared to generate a standard curve. A four parameter logistic curve fit was selected to generate the standard curve and calculate the concentration of each sample.

### Real-time quantitative PCR

The distal ileum and liver tissues were homogenized using TissueLyzer (QIAGEN) and total RNA was isolated using TRIzol Reagent (Invitrogen, Life Technology, USA). The total RNA concentration was measured using a NanoDrop 2000C spectrophotometer (Thermo Fisher Scientific, Waltham, MA,USA). A purified, 500 ng sample of total RNA from each ileal/liver sample were reverse transcribed using random hexamer primers to form the cDNA templates employing a Prime Script RT Reagent Kit (TAKARA, Kusatsu, Japan). The qPCR primers were designed and synthesized (Sangon Biotech,Shanghai, China) and the forward and reverse sequences are shown in Supplementary table 1. The quantitative real-time PCR reaction mixture was set up using Power Up SYBR Green PCR Master Mix (Applied Biosystems, Thermo Fisher Scientific, USA) and the reaction was run in an ABI 7900HT Real-Time PCR System (Applied Biosystems Instruments, Thermo Fisher Scientific, USA). All the procedures were handled following the manufacturer’s instructions. The values of the target genes were normalized to GAPDH and the relative expression level were shown as fold changes relative to control group.

### Western blot analysis

Ileum, liver and cell samples were lysed with RIPA buffer (Beyotime Technology, Shanghai, China) containing 1 mM PMSF (Beyotime Technology, Shanghai, China) in an ice bath followed by centrifugation at 14,000 g for 5 min. The supernatants were collected and protein concentrations were measured using a BCA Protein Assay Kit (Pierce, Rockford, IL, USA). Specifically, the nuclear protein that was separated using a nuclear and cytoplasmic separation reagent (NE-PER, Thermo Scientific, 78833) according to the manufacturing instructions for western blot analysis of FXR. A 5 μg/μL of protein extract was supplied with loading buffer (Beyotime Technology, Shanghai, China) and denatured by boiling at 100 °C for 10 min. The denatured proteins were resolved by 12% SDS-PAGE, and transferred to Immobilon-P Transfer Membranes (Millipore Corporation, Tullagreen, IRL). The membranes were blocked with 5% BSA (Beyotime Technology, Shanghai, China) at room temperature for 1 h, incubated with primary antibodies over night at 4 °C, and then incubated with horseradish peroxidase conjugated secondary antibodies. The bands were visualized using a SuperSignal West Pico Chemiluminescent Substrate (Thermo Scientific, Rockford, IL, USA) with a Tanon 5,500 Chemiluminescent Imaging System (Tanon Science & Technology Co., Shanghai, China). The gray values of the bands were calculated using ImageJ software and were normalized to β-Actin. The antibodies used for mouse tissue and the antibody dilutions were as follows: 1:500 for rabbit anti-FXR (Abcam, Cambridge, MA), 1:1000 for mouse anti-FGF15 (Santa Cruze Biotechnology, USA), 1:900 for rabbit anti-FGF19 (Abcam, Cambridge, MA), 1:1000 for rabbit anti-CYP7A1 (Abcam, Cambridge, MA), 1:1,000 for rabbit anti-CYP8B1 (Abcam, Cambridge, MA), 1:10,000 for rabbit anti-CYP27A1 (Abcam, Cambridge, MA), 1:10000 for rabbit anti-CYP7B1 (Abcam, Cambridge, MA), 1:2000 for rabbit anti-β-Actin (Cell Signaling Technology, MA), 1:1000 for rabbit anti-Lamin B1 (Beyotime Biotechnology, China). The nuclear and cytoplasmic protein that was separated using a nuclear and cytoplasmic seperation reagent (NE-PER, Thermo Scientific, 78833) according to the manufacturer’s instructions for western blot analysis of FXR.

### Preparation and culture of human FHs 74 Int and L02 cells

Human intestine FHs 74 Int and liver L02 cell lines were purchased from American Type Culture Collection (ATCC) and Type Culture Collection of Chinese Academy of Science respectively, cultured in Hybri-Care or RPMI-1640 medium supplemented with 10% fetal bovine serum (FBS) (Omega Scientific, Tarzana, CA) and then incubated at 37 °C in a humidified atmosphere containing 5% CO_2_ in air. The FHs 74 Int and L02 cells were treated with TCA (50 μM), CDCA (50 μM) alone or with TUDCA (50 μM), TCDCA (50 μM), respectively for 24 h. The L02 cells were treated with different concentrations of FGF19 (25, 50, 75 and 100 ng/mL) for 48 h. Cell samples were then harvested for immunofluorescence staining and western blot protein analysis including FXR and FGF19 in intestine FHs 74 Int and L02 cells and CYP7A1, CYP8B1, CYP27A1, CYP7B1 in liver L02 cell.

### Liver immunohistochemistry analysis

Liver tissues were fixed with 4% paraformaldehyde solution, embedded in paraffin blocks and processed by immunohistochemistry staining. Tissue sections were deparaffinized and rehydrated using a graded ethanol series and distilled water, and then treated with 3% H_2_O_2_ in methanol for 30 min to block endogenous peroxidase activity. Tissue sections were then rinsed twice for five minutes in phosphate-buffered saline (PBS) and incubated with 10% normal goat serum for 30 min to block non-specific antibody binding. After washing, the samples were incubated with primary antibodies against CYP7A1 (Abcam, ab234982, 1:500), CYP8B1 (Abcam, ab175843, 1:50), CYP27A1 (Abcam, ab126785, 1:250), and CYP7B1 (Abcam, ab175889, 1:100). Sections were then washed in PBS three times and incubated with secondary antibodies. The sections were stained with DAB according to the manufacturer’s protocol, mounted on slides, and photographed using a digital microscope camera (Nikon, Tokyo, Japan). The immunohistochemistry sample images were quantified using Image-Pro Plus software (Media Cybernetics, MD, USA).

### Statistical analysis

Raw data from BA and Tea component quantification were obtained with MassLynx v4.1 and analyzed by TargetLynex v4.1 (Waters, Milford, MA). All the bar plots in this study were generated with GraphPad Prism 6.0 (GraphPad Software, San Diego, USA), and differential analysis using the Mann-Whitney U test or Wilcoxon rank-sum test was conducted using SPSS 20.0 (IBM SPSS, USA) with significant criteria set to be *p-value < 0.05 and #*p*-value < 0.005. Sequence data analyses for 16S rRNA gene sequencing analysis were performed using QIIME and R packages (v3.2.0). OTU-level alpha diversity indices, such as Chao1 richness estimator, ACE metric (Abundance-based Coverage Estimator), Shannon diversity index, and Simpson index, were calculated using the OTU table in QIIME. OTU-level ranked abundance curves were generated to compare the richness and evenness of OTUs among samples. Beta diversity analysis was performed to investigate the structural variation of microbial communities across samples using UniFrac distance metrics and visualized via principal coordinate analysis (PCoA). LEfSe was performed to detect differentially abundant taxa across groups using the default parameters. Differences in the Unifrac distances for pairwise comparisons among groups were determined using Student’s *t*-test and the Monte Carlo permutation test with 1000 permutations. The taxonomy compositions and abundances were visualized using MEGAN and GraPhlAn. The phylogenetic tree that clustered the differential OTUs was constructed and visualized using MEGA (v7.0.26).

### Reporting summary

Further information on research design is available in the Nature Research Reporting Summary linked to this article.

## Supplementary information

Supplementary Information

Reporting Summary

Source Data

## Data Availability

The source data underlying Figs. 1–7 and Supplementary Figs. 2–14 are provided as a Source Data file. The 16S rRNA gene sequences and metagenomic sequences were provided and available at NCBI Sequence Read Archive (SRP) repository with accession code SRP221307, SRP221311, SRP221451, SRP221525, SRP221313, SRP221862. The metabolomics data were deposited and available at Metabolights repository with accession code MTBLS1259. Other data supporting the findings of this study are available from the corresponding authors upon reasonable request.
