# Supplementary material for: Theabrownin from Pu-erh tea attenuates hypercholesterolemia via modulation of gut microbiota and bile acid metabolism
Source: Nat Commun. 2019 Oct 31;10:4971. doi: 10.1038/s41467-019-12896-x (PMC6823360; doi:10.1038/s41467-019-12896-x)
Supplement: Supplementary file 1 — Supplementary Information [file 41467_2019_12896_MOESM1_ESM.pdf]

Supplementary Information

Theabrownin from Pu-erh tea attenuates hypercholesterolemia via modulation of gut microbiota and bile acid metabolism

Huang et al.

Supplementary Figures

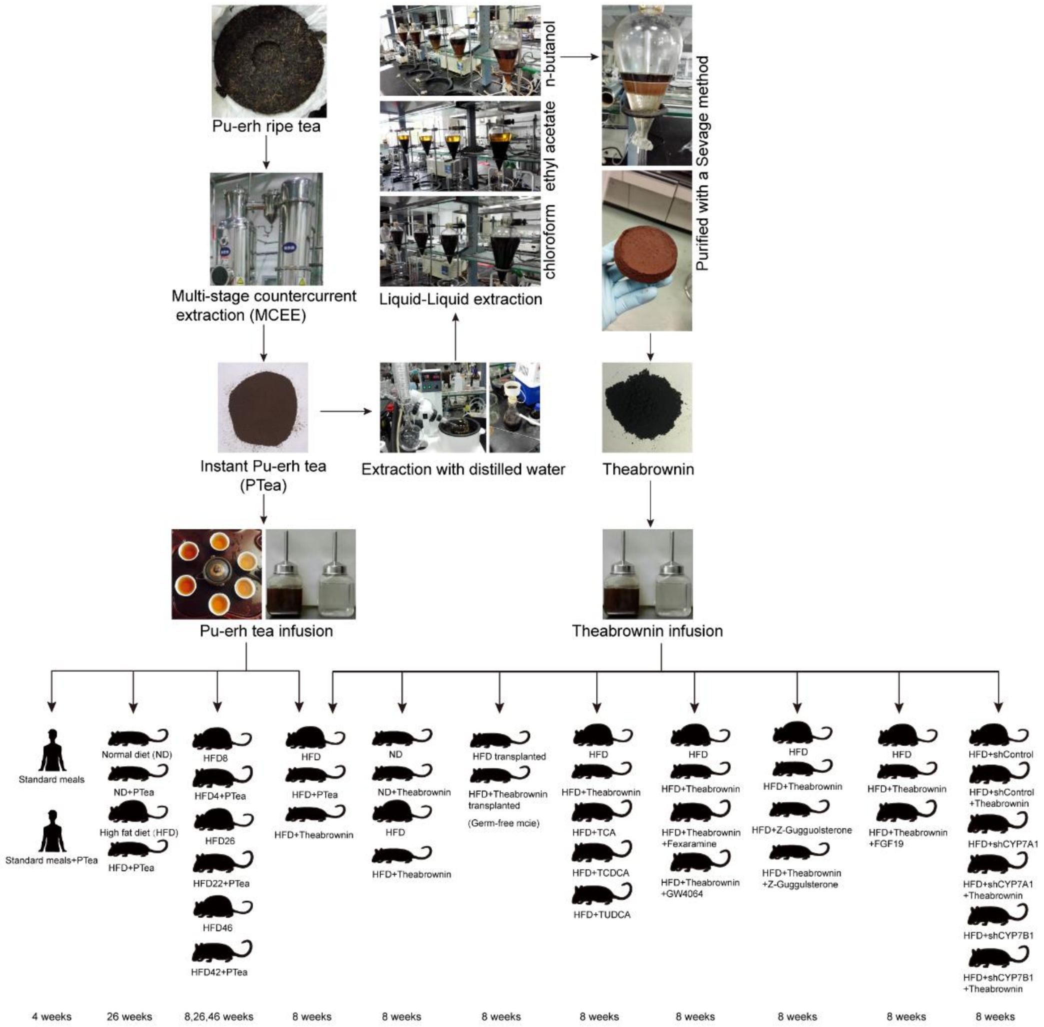

Supplementary Fig. 1. Animal experimental design.

Flow chart of instant Pu-erh tea and theabrownin production and experimental use on mice fed normal diet or HFD and on human subjects that received standard meals. Instant Pu-erh tea was extracted from Pu-erh ripe tea using a multi-stage countercurrent extraction (MCEE) method. The theabrownin was extracted from instant Pu-erh tea using distilled water, followed by a series of liquid-liquid extractions with chloroform, ethyl acetate, n-butanol, respectively, and subsequently purified using a Sevage method. A total of 11 experiments using extracted Pu-erh tea and theabrownin in human subjects and mice were conducted as follows:

Experiment 1, human subjects received standard meals without Pu-erh tea for one week (Pre-PTea), and subsequently were supplied with standard meals and 50 mg/Kg/day Pu-erh tea for 4 weeks (Post-PTea);

Experiment 2, a) mice received normal diet for 26 weeks (ND), b) mice received a normal diet and were supplied with 450 mg/Kg/day Pu-erh tea for 26 weeks (ND+PTea), c) mice received high fat diet for 26 weeks (HFD), d) mice received high fat diet and were supplied with 450 mg/Kg/day Pu-erh tea for 26 weeks (HFD+PTea);

Experiment 3, a) mice received a HFD for 8 weeks (HFD8), b) mice received a HFD for 4 weeks and were further supplied with 450 mg/Kg/day Pu-erh tea for 4 weeks (HFD4+PTea), c) mice received a HFD for 26 weeks (HFD26), d) mice received a HFD for 22 weeks and were further supplied with 450 mg/Kg/day Pu-erh tea for 4 weeks (HFD22+PTea), e) mice received a HFD for 42 weeks (HFD42), f) mice received a HFD for 42 weeks and were further supplied with 450 mg/Kg/day Pu-erh tea for 4 weeks (HFD42+PTea);

Experiment 4, a) mice received a high fat diet for 8 weeks(HFD), b) mice received high fat diet and were supplied with 450 mg/Kg/day Pu-erh tea for 8 weeks (HFD+PTea), c) mice received a high fat diet and were supplied with 225 mg/Kg/day theabrownin for 8 weeks (HFD+Theabrownin);

Experiment 5, a) mice received normal diet for 8 weeks (ND), b) mice received a normal diet and were supplied with 225 mg/Kg/day theabrownin tea for 8 weeks (ND+Theabrownin), c) mice received a HFD for 8 weeks (HFD), d) mice received a HFD and were supplied with 225 mg/Kg/day theabrownin tea for 8 weeks (HFD+Theabrownin);

Experiment 6, a) germ-free mice transplanted with microbiota from mice received a high fat diet (HFD), b) germ-free mice transplanted with microbiota from mice received a HFD and 225 mg/Kg/day theabrownin (HFD+Theabrownin);

Experiment 7, a) mice received high fat diet for 8 weeks (HFD), b) mice received a high fat diet and were supplied with 225 mg/Kg/day theabrownin for 8 weeks (HFD+Theabrownin), c) mice that received high fat diet and were supplied with 50 mg/Kg/day TCA for 8 weeks (HFD+TCA), d) mice that received high fat diet and were supplied with 50 mg/Kg/day TCDCA for 8 weeks (HFD+TCDCA), e) mice that received high fat diet and were supplied with 50 mg/Kg/day TUDCA for 8 weeks (HFD+TUDCA);

Experiment 8, a) mice that received high fat diet for 8 weeks (HFD), b) mice that received high fat diet and were supplied with 225 mg/Kg/day theabrownin for 8 weeks (HFD+Theabrownin), c) mice that received a high fat diet and were supplied with 225 mg/Kg/day theabrownin and 100 mg/Kg/day fexaramine for 8 weeks (HFD+Theabrownin+Fexaramine), d) mice that received a high fat diet and were supplied with 225 mg/Kg/day theabrownin and 100 mg/Kg/day GW4064 for 8 weeks (HFD+Theabrownin+GW4064);

Experiment 9, a) mice that received a HFD for 8 weeks (HFD), b) mice that received a HFD and were supplied with 225 mg/Kg/day theabrownin for 8 weeks (HFD+Theabrownin), c) mice that received a HFD and were supplied with 100 mg/Kg/day Z-Guggulsterone for 8 weeks (HFD+Z-guggulsterone), d) mice that received a HFD and were supplied with 225 mg/Kg/day theabrownin and 100 mg/Kg/day Z-Guggulsterone for 8 weeks (HFD+Theabrownin+ Z-Guggulsterone);

Experiment 10, a) mice that received high fat diet for 8 weeks (HFD), b) mice that received high fat diet and were supplied with 225 mg/Kg/day theabrownin for 8 weeks (HFD+Theabrownin), c) mice that received high fat diet and were supplied with 225 mg/Kg/day theabrownin and 50 μg/Kg/day recombinant FGF19 protein for 8 weeks (HFD+Theabrownin+FGF19);

Experiment 11, a) mice received shControl adeno-associated virus (AAV) were fed a HFD for 8 weeks (HFD+shControl), b) mice received shControl AAV were fed a HFD and supplied with 225 mg/Kg/day for 8 weeks (HFD+shControl+Theabrownin), c) mice received shCYP7A1 AAV were fed a HFD for 8 weeks (HFD+shCYP7A1), d) mice received shCYP7A1 AAV were fed a HFD and supplied with 225 mg/Kg/day for 8 weeks (HFD+shCYP7A1+Theabrownin), e) mice received shCYP7B1 AAV were fed a HFD for 8 weeks (HFD+shCYP7B1), f) mice received shCYP7B1 AAV were fed a HFD and supplied with 225 mg/Kg/day for 8 weeks (HFD+shCYP7B1+Theabrownin).

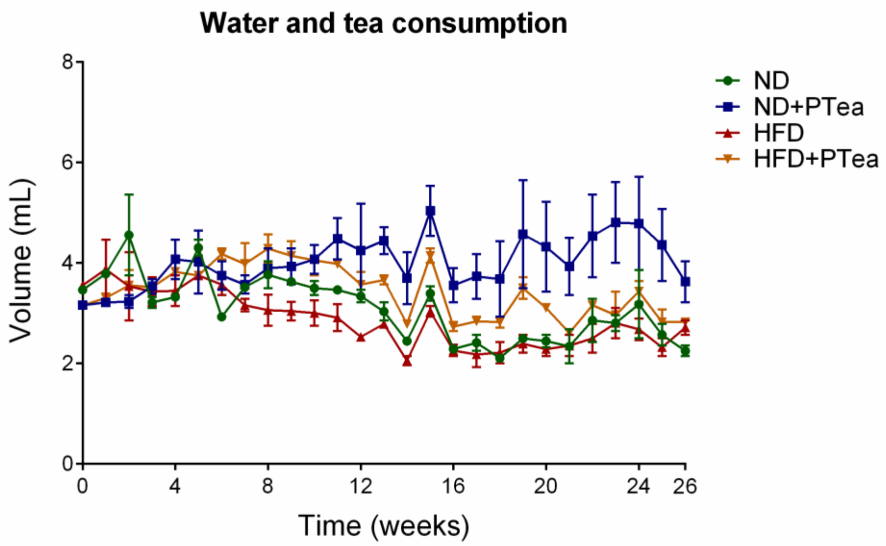

Supplementary Fig. 2. Consumption of water and Pu-erh tea infusion by mice. The intake volume of water and tea infusion by ND or HFD fed mice after 26 weeks of the tea intervention. n=8 individuals/group. Data were calculated as the drinking volume per mouse per day and expressed as mean ±SEM.

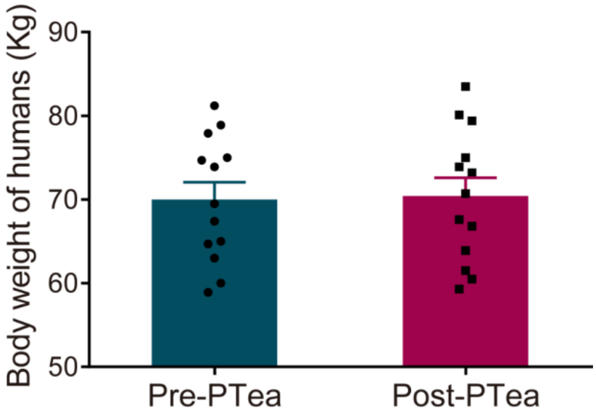

Supplementary Fig. 3. The body weight of human subjects before and after Pu-erh tea consumption.

The body weight of human subjects before (Pre-PTea) and after (Post-PTea) 4 weeks of Pu-erh tea consumption was not significantly changed. n=13 individuals/group. Data were expressed as mean±SEM. Differences of data in human subjects were assessed by Wilcoxon rank-sum test, * $p<0.05$, # $p<0.005$.

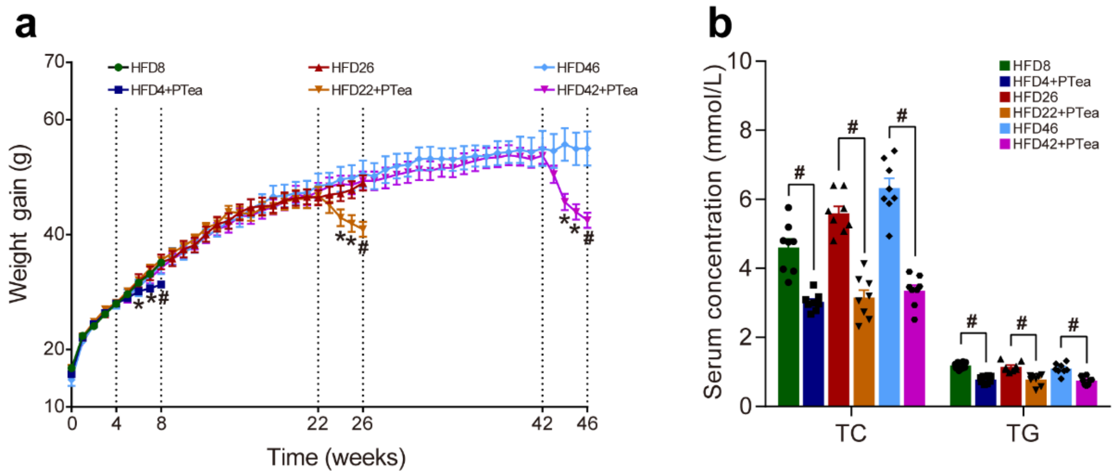

Supplementary Fig. 4. The body weight and lipid lowering effects of Pu-erh tea on HFD-induced obesity.

a Pu-erh tea reduced the body weight of mice fed with a HFD for 4, 22 and 42 weeks. **b** Pu-erh tea reduced serum TC and TG concentration of mice fed a HFD for 4, 22 and 42 weeks. HFDn: mice treated with a HFD for n weeks (n=4, 8, 22, 26, 42, and 46 weeks), PTea: mice treated with Pu-erh tea for 4 weeks. n=8 individuals/group. Data were expressed as mean ± SEM. Data in HFD8, HFD26 and HFD46 groups were compared to HFD4+PTea, HFD22+PTea and HFD42+PTea groups respectively. Differences of data were assessed by Mann-Whitney U test, * p<0.05, # p<0.005.

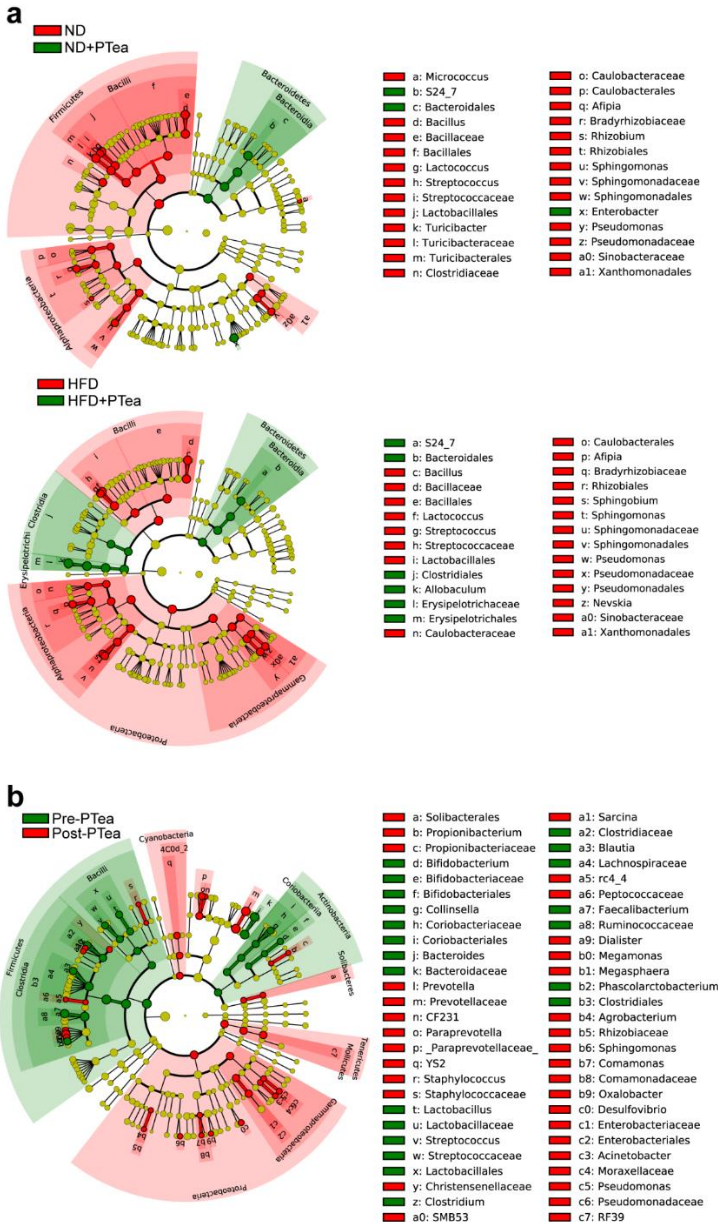

Supplementary Fig. 5. LEfSe analysis on microbes in mice ileum and human feces on Pu-erh tea consumption.

a Pu-erh tea induced differential microbes, identified by LEfSe analysis, at different phylogenetic levels in mouse ileum. n=5 individuals/group. **b** Pu-erh tea induced differential microbes, identified by LEfSe analysis, at different phylogenetic levels in human feces. n=13 individuals/group. LEfSe were conducted on ND to ND+PTea, HFD to HFD+PTea and Pre-PTea to Post-PTea.

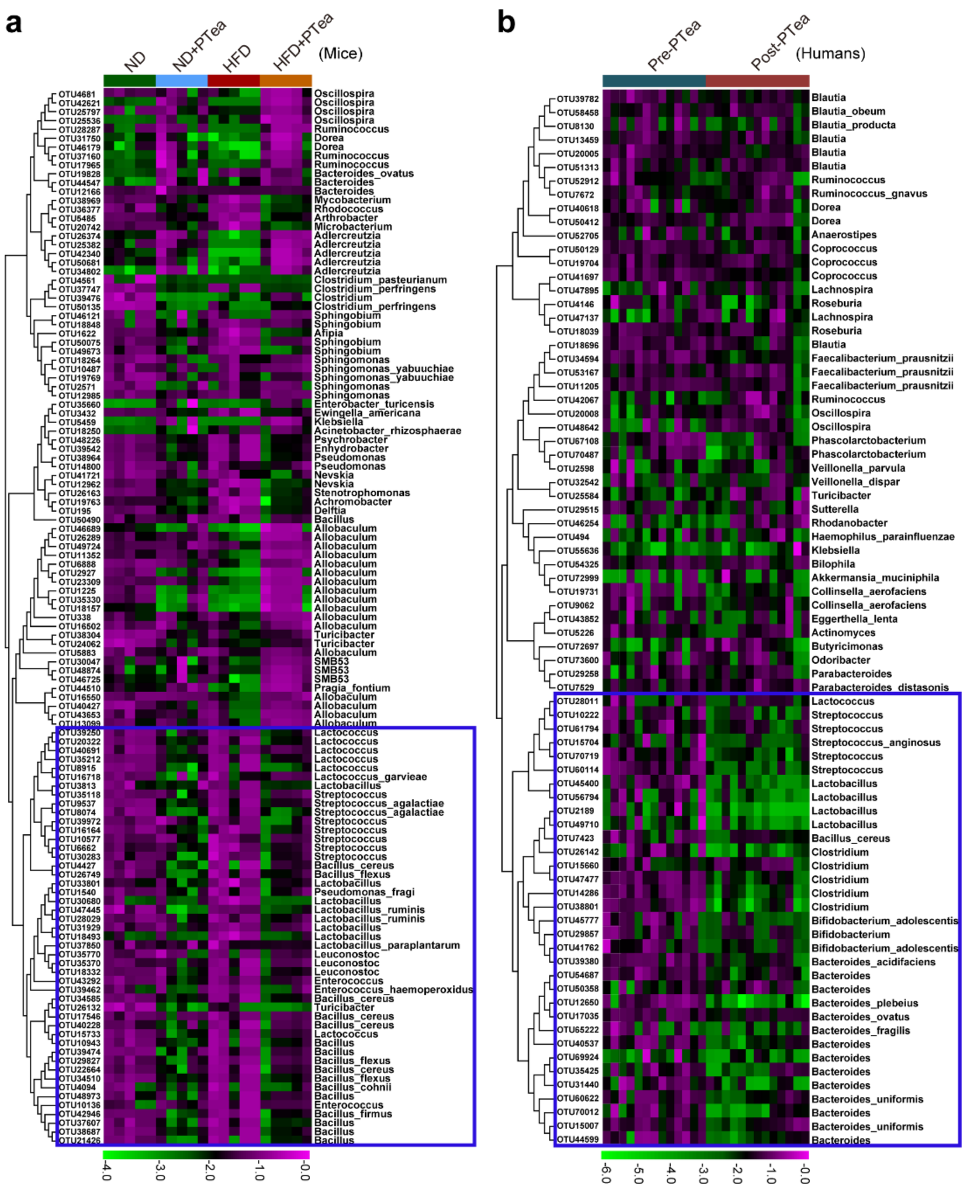

Supplementary Fig. 6. Relative abundance of representative OTUs at genus and species in mice and human subjects on Pu-erh tea consumption.

a Heatmap of the relative abundance of representative OTUs at the genus and species levels in mice. n=5 individuals/group. **b** Heatmap of the relative abundance of representative OTUs at the genus and species levels in human subjects. n=13 individuals/group. The color of each spot in the heatmap corresponds to the normalized and log-transformation of the raw abundance of the OTUs in each sample. The OTUs were organized according to their order in a phylogenetic tree generated by their representative sequences.

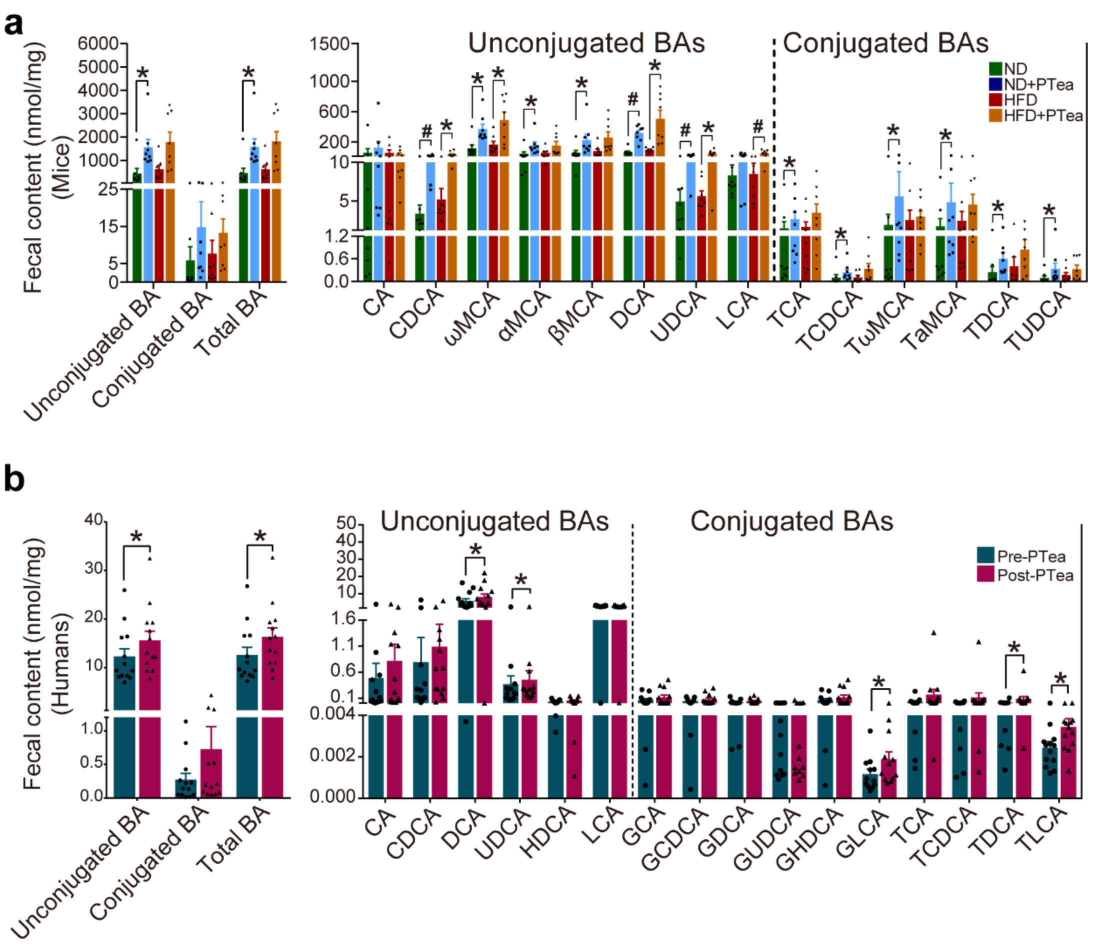

Supplementary Fig. 7. Changes of fecal BA profiles of mice and human subjects induced by Pu-erh tea.

a Fecal BA classes and BA profiles of mice after Pu-erh tea consumption for 26 weeks on both normal and HFD. n=8 individuals/group. **b** Fecal BA classes and BA profiles of human subjects before and after Pu-erh tea consumption after 4 weeks on a standard meal. n=13 individuals/group. Data were expressed as mean±SEM. Differences of data for mice and human subjects were assessed using the Mann-Whitney U test and Wilcoxon rank-sum test, respectively, * $p<0.05$, # $p<0.005$.

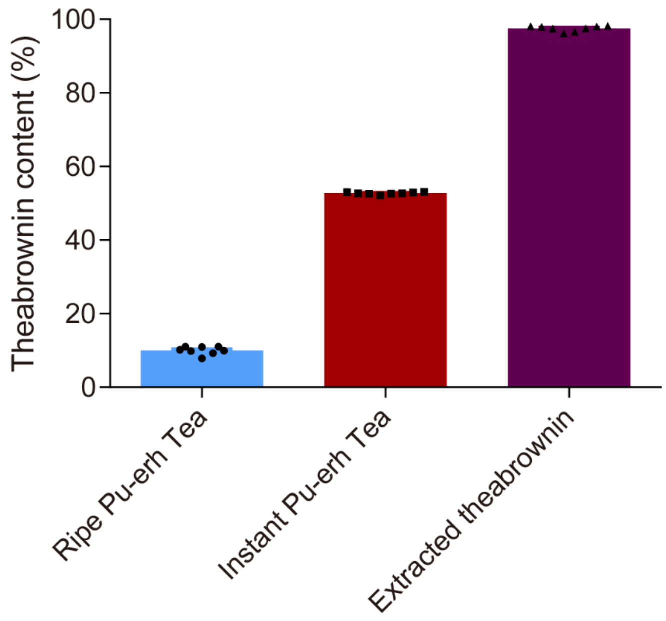

Supplementary Fig. 8. Comparison on the content of theabrownin in ripe Pu-erh tea, instant Pu-erh tea and extracted theabrownin.

The average percentage content of theabrownin in ripe Pu-erh tea, instant Pu-erh tea and extracted theabrownin were 10.03%, 52.75% and 97.51% respectively. n=8 individuals/group. The content in extracted theabrownin was much higher than in either ripe Pu-erh tea or instant Pu-erh tea.

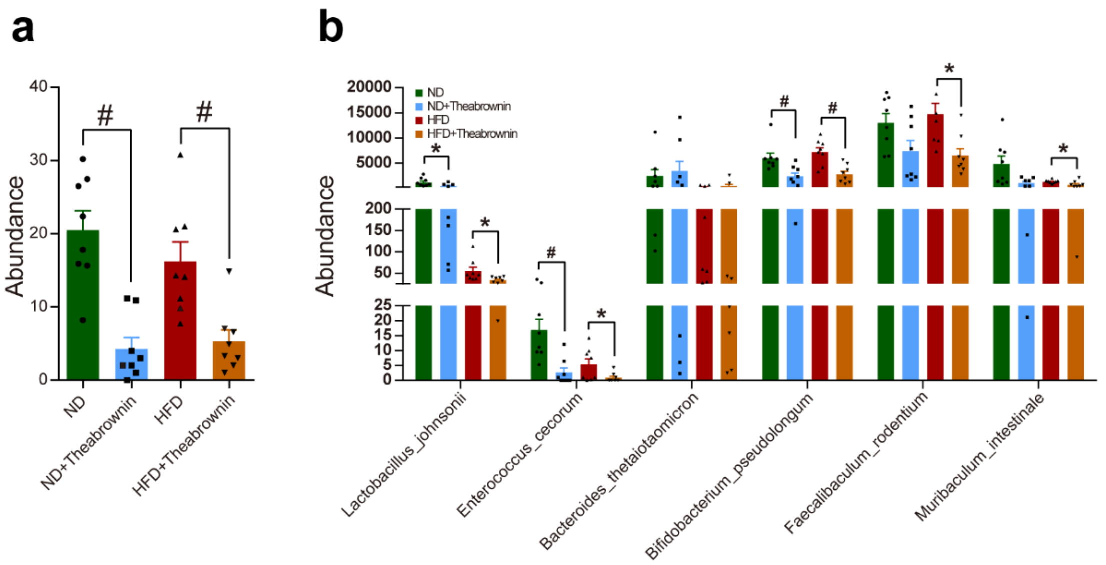

Supplementary Fig. 9. Theabrownin significantly reduced the BSH function and abundance of BSH microbiota at the species level.

a BSH function in ileum of ND and HFD fed mice treated with 225 mg/Kg/day theabrownin for 8 weeks. n=8 individuals/group. **b** Abundance of BSH related microbiota at the species level in ileum of ND and HFD fed mice treated with 225 mg/Kg/day theabrownin for 8 weeks. n=8 individuals/group. Data were expressed as mean±SEM. Differences were assessed using the Mann-Whitney U test, * p<0.05, # p<0.005.

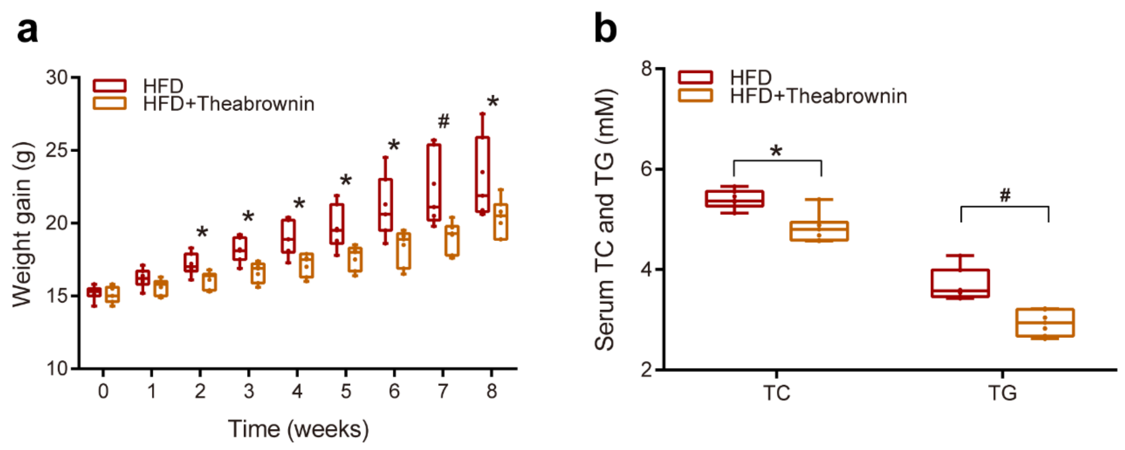

Supplementary Fig. 10. Body weight and serum lipids level of germ-free mice transplanted with microbiota from mice treated with HFD and theabrownin.

a Body weight of germ-free mice transplanted with microbiota from control and theabrownin treated mice. n=8 individuals/group. **b** Serum TC and TG level of germ-free mice transplanted with microbiota from control and theabrownin treated mice. n=8 individuals/group. Differences of data were assessed by Mann-Whitney U test, * $p<0.05$, # $p<0.005$.

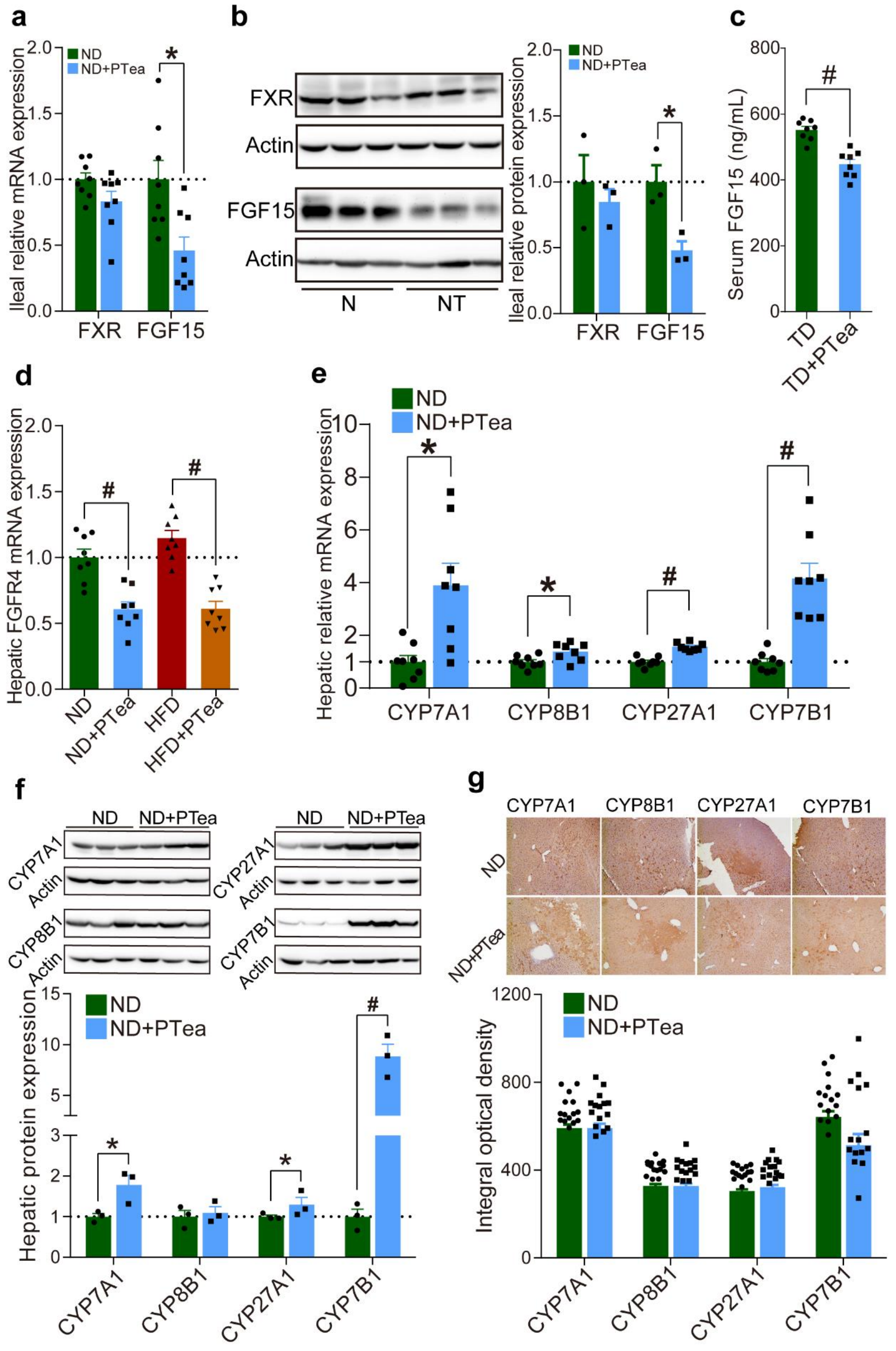

Supplementary Fig. 11. Conjugated BAs inhibit the gut-liver FXR-FGF15 signaling pathway to induce hepatic BA synthetic enzyme expression.

a Gene expression of FXR and FGF15 in the distal ileum for mice fed normal diet along with consumption of 450 mg/Kg/day Pu-erh tea for 26 weeks. n=8 individuals/group. **b** Relative protein expression levels of FXR and FGF15 in the distal ileum for mice fed normal diet along with consumption of 450 mg/Kg/day Pu-erh tea for 26 weeks. n=3 individuals/group. **c** Serum FGF15 concentration for mice fed normal diet along with consumption of 450 mg/Kg/day Pu-erh tea for 26 weeks. n=8 individuals/group. **d** The hepatic FGFR4 mRNA expression for normal and HFD fed mice that consumed 450 mg/Kg/day Pu-erh tea for 26 weeks. n=8 individuals/group. **e** Expression of hepatic BA synthetic genes CYP7A1, CYP8B1, CYP27A1 and

CYP7B1 for mice fed normal diet along with consumption of 450 mg/Kg/day Pu-erh tea for 26 weeks. n=8 individuals/group. **f** Relative protein expression levels of BA synthetic genes CYP7A1, CYP8B1, CYP27A1 and CYP7B1 in liver for mice fed normal diet along with consumption of 450 mg/Kg/day Pu-erh tea for 26 weeks. n=3 individuals/group. **g** Relative protein expression levels of BA synthetic genes CYP7A1, CYP8B1, CYP27A1 and CYP7B1identified by IHC histological staining in liver for mice fed normal diet along with consumption of 450 mg/Kg/day Pu-erh tea for 26 weeks. Data were expressed as mean±SEM. Differences of data were assessed using the Mann-Whitney U test, * p<0.05, # p<0.005.

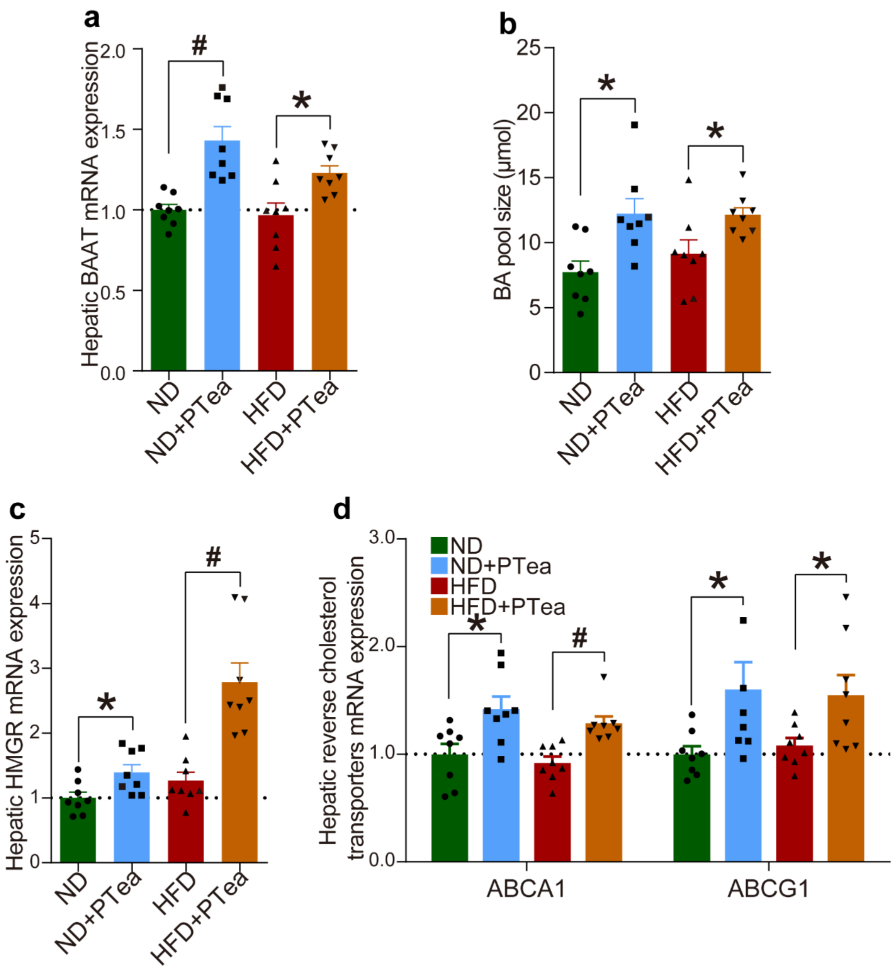

Supplementary Fig. 12. Expression of genes related to BA and cholesterol metabolism along with the bile acid pool size affected by Pu-erh tea.

a Gene expression of hepatic BAAT in normal diet and HFD fed mice that consumed 450 mg/Kg/day Pu-erh tea for 26 weeks. n=8 individuals/group. **b** The BA pool size in normal diet and HFD fed mice that consumed 450 mg/Kg/day Pu-erh tea for 26 weeks. The BA pool size refers to the total amount of BAs in the enterohepatic circulation which consists of serum, liver, small intestine and contents, cecum and contents, colon and contents, and feces. n=8 individuals/group. **c** Gene expression of hepatic HMGR in normal diet and HFD fed mice consumed 450 mg/Kg/day Pu-erh tea for 26 weeks. n=8 individuals/group. **d** Gene expression of hepatic reverse cholesterol transporters (RCT) ABCA1 and ABCG1in normal diet and HFD fed mice consumed 450 mg/Kg/day Pu-erh tea for 26 weeks. n=8 individuals/group. Data were expressed as mean±SEM. Differences of data were assessed using the Mann-Whitney U test, * p<0.05, # p<0.005.

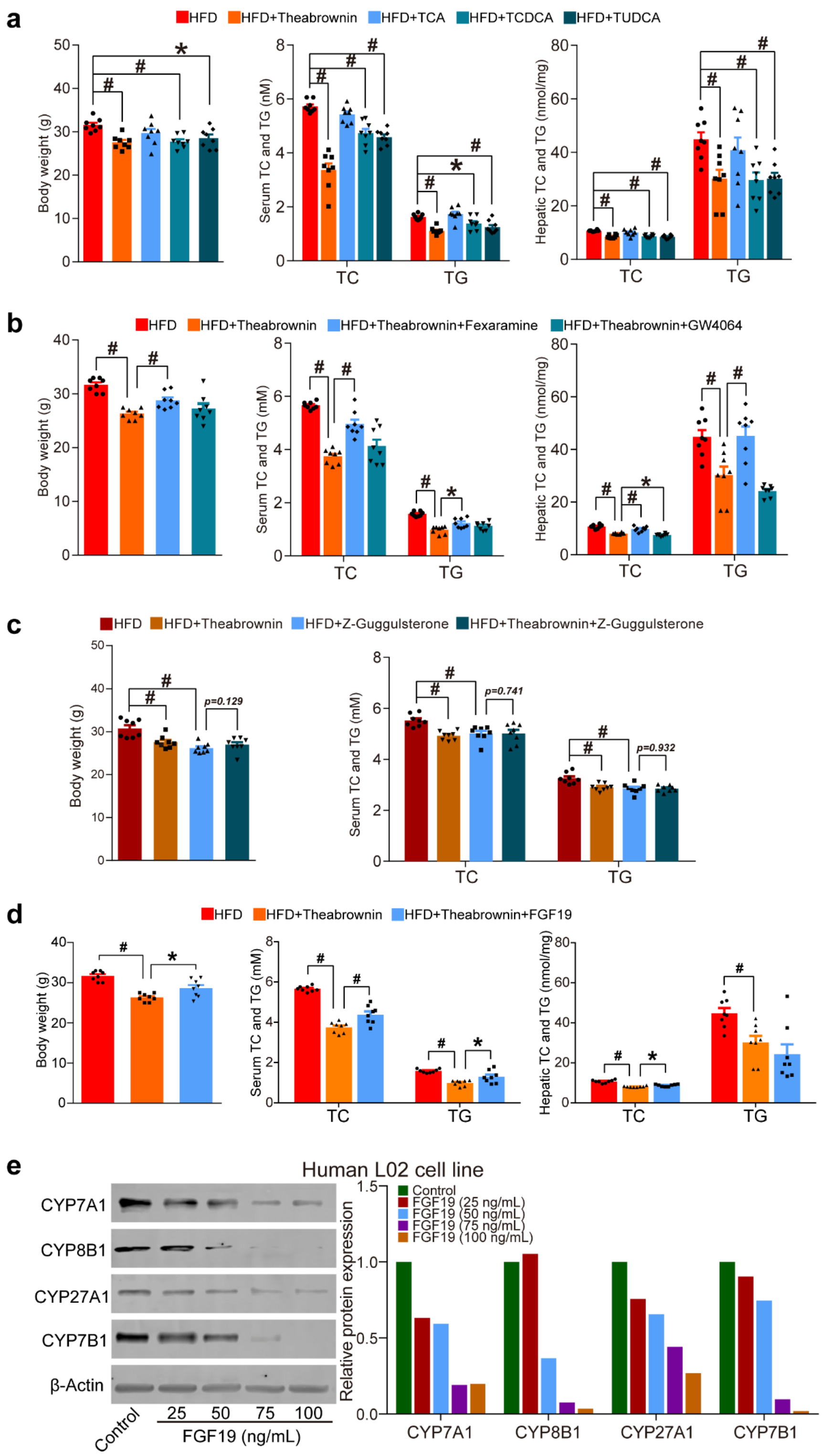

Supplementary Fig. 13. Modulation of ileal FXR-FGF15 signaling sensitively regulated hepatic BA synthesis and cholesterol levels.

a Cholesterol levels and body weights of HFD fed mice supplied with 225 mg/Kg/day theabrownin, 50 mg/Kg/day TCDCA and 50 mg/Kg/day TUDCA for 8 weeks. n=8 individuals/group. **b** Cholesterol levels and body weights of HFD fed mice supplied with 225 mg/Kg/day theabrownin, theabrownin coupled with 100 mg/Kg/day fexaramine for 8 weeks. n=8 individuals/group. **c** Cholesterol levels and body weights

of HFD fed mice supplied with 225 mg/Kg/day theabrownin, 100 mg/Kg/day Z-Guggulsterone, or 225 mg/Kg/day theabrownin coupled with 100 μg/Kg/day Z-Guggulsterone for 8 weeks. n=8 individuals/group. **d** Cholesterol levels and body weights of HFD fed mice gavaged with 225 mg/Kg/day theabrownin and also supplied with 225 mg/Kg/day theabrownin coupled with 50 μg/Kg/day recombinant FGF19 protein by intraperitoneal injection for 8 weeks. n=8 individuals/group. **e** FGF19 reduced expression of BA synthetic gene expression in the human L02 cell line. Protein expression was normalized with β-Actin. Data were expressed as mean±SEM. Differences between data were assessed by the Mann-Whitney U test, * $p<0.05$, # $p<0.005$.

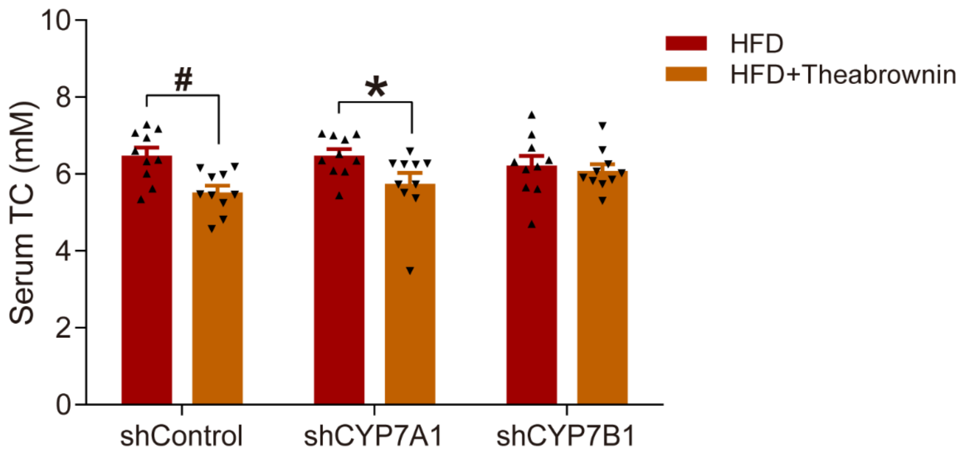

Supplementary Fig. 14. Theabrownin regulated serum TC level in AAV-shRNA knockdown of hepatic CYP7A1 and CYP7B1 mice.

Serum TC level of hepatic CYP7A1 and CYP7B1 AAV-shRNA knockdown mice treated with 225 mg/Kg/day theabrownin for 8 weeks. n=8 individuals/group. Data were expressed as mean±SEM. Differences of data were assessed using the Mann-Whitney U test, * $p<0.05$, # $p<0.005$.

Supplementary Table

Supplementary Table 1. Primers for qPCR

Gene name	Forward sequence (5' to3')	Reverse sequence (5' to3')
FXR	CCCCTGCTTGATGTGCTAC	CGTGGTGATGGTTGAATGTC
FGF15	TGTTTCACCGCTCCTTCTTT	TCTACATCCTCCACCATCCTG
CYP7A1	CTGGGCTGTGCTCTGAAGT	GGGAGTTTGTGATGAAGTGGA
CYP8B1	ACAGCGTGATGGAGGAGAGT	AGGGGAAGAGAGCCACCTTA
CYP27A1	TCCCAGTGTCTTTCCTGAGC	CACAGAGCCGAATGGATGTA
CYP7B1	TGAGGTTCTGAGGCTGTGC	TGGAGGAAAGAGGGCTACAA
FGFR4	GCATCTTTCAGGGGACACCA	TTGTACCAGTGACGACCACG
BAAT	TAGCCCCTACCAAATCCACA	GCCACATACCACCTTTCCAG
HMGR	TGCCTTGTGATTGGAGTTGG	TAGGACCAGCGACACACAGG
ABCA1	TCTGATGACCACCTCTGTCG	GAAGATGCTTGGCTTTGCTC
ABCG1	CACCCAGTTCTGCATCCTCT	AGGTACAGCAGGCCAATGAG
