# Supplementary material for: Theabrownin from Pu-erh tea attenuates hypercholesterolemia via modulation of gut microbiota and bile acid metabolism
Source: Nat Commun. 2019 Oct 31;10:4971. doi: 10.1038/s41467-019-12896-x (PMC6823360; doi:10.1038/s41467-019-12896-x)
Supplement: Supplementary file 2 — Reporting Summary [file 41467_2019_12896_MOESM2_ESM.pdf]

nature research

Corresponding author(s): Wei Jia, Aihua Zhao, Houkai Li

Last updated by author(s): Sep 22, 2019

Reporting Summary

Nature Research wishes to improve the reproducibility of the work that we publish. This form provides structure for consistency and transparency in reporting. For further information on Nature Research policies, see Authors & Referees and the Editorial Policy Checklist.

Statistics

For all statistical analyses, confirm that the following items are present in the figure legend, table legend, main text, or Methods section.

n/a	Confirmed	
☐	☒	The exact sample size (*n*) for each experimental group/condition, given as a discrete number and unit of measurement
☐	☒	A statement on whether measurements were taken from distinct samples or whether the same sample was measured repeatedly
☐	☒	The statistical test(s) used AND whether they are one- or two-sided<br>*Only common tests should be described solely by name; describe more complex techniques in the Methods section.*
☒	☐	A description of all covariates tested
☒	☐	A description of any assumptions or corrections, such as tests of normality and adjustment for multiple comparisons
☐	☒	A full description of the statistical parameters including central tendency (e.g. means) or other basic estimates (e.g. regression coefficient) AND variation (e.g. standard deviation) or associated estimates of uncertainty (e.g. confidence intervals)
☐	☒	For null hypothesis testing, the test statistic (e.g. *F*, *t*, *r*) with confidence intervals, effect sizes, degrees of freedom and *P* value noted<br>*Give P values as exact values whenever suitable.*
☒	☐	For Bayesian analysis, information on the choice of priors and Markov chain Monte Carlo settings
☐	☒	For hierarchical and complex designs, identification of the appropriate level for tests and full reporting of outcomes
☐	☒	Estimates of effect sizes (e.g. Cohen's *d*, Pearson's *r*), indicating how they were calculated

Our web collection on statistics for biologists contains articles on many of the points above.

Software and code

Policy information about availability of computer code

Data collection	MassLynx v4.1 and TargetLynex v4.1 (Waters, Milford, MA) were used for data collection in the manuscript
Data analysis	GraphPad Prism 6.0 (GraphPad Software, San Diego, USA), SPSS 20.0 (IBM SPSS, USA) were used for statistical analyses and graphic generation.

For manuscripts utilizing custom algorithms or software that are central to the research but not yet described in published literature, software must be made available to editors/reviewers. We strongly encourage code deposition in a community repository (e.g. GitHub). See the Nature Research guidelines for submitting code & software for further information.

Data

Policy information about availability of data

All manuscripts must include a data availability statement. This statement should provide the following information, where applicable:

- Accession codes, unique identifiers, or web links for publicly available datasets
- A list of figures that have associated raw data
- A description of any restrictions on data availability

The data that support the findings of this study are available from the corresponding author upon reasonable request. The source data underlying Figs. 1-7 and Supplementary Figs. 2-14 are provided as a Source Data file. The 16S rRNA gene sequences and metagenomic sequences were provided and available at NCBI Sequence Read Archive (SRP) repository with accession code SRP221307 [https://trace.ncbi.nlm.nih.gov/sra/?term=SRP221307],SRP221311[https://trace.ncbi.nlm.nih.gov/sra/?term=SRP221311],SRP221451[https://trace.ncbi.nlm.nih.gov/sra/?term=SRP221451],SRP221525[https://trace.ncbi.nlm.nih.gov/sra/?term=SRP221525],SRP221313[https://trace.ncbi.nlm.nih.gov/sra/?term=SRP221313],SRP221862[https://trace.ncbi.nlm.nih.gov/sra/?term=SRP221862]. The metabolomics data were deposited and available at Metabolights repository with accession code MTBLS1259 [www.ebi.ac.uk/metabolights/MTBLS1259].

Field-specific reporting

Please select the one below that is the best fit for your research. If you are not sure, read the appropriate sections before making your selection.

☒ Life sciences ☐ Behavioural & social sciences ☐ Ecological, evolutionary & environmental sciences

For a reference copy of the document with all sections, see nature.com/documents/nr-reporting-summary-flat.pdf

Life sciences study design

All studies must disclose on these points even when the disclosure is negative.

Sample size	Sample sizes of all studies were predetermined by the software GPower before experiments. The sample sizes are all larger than the predetermined ones when the powers are set as 0.85 (significant level 0.05, two tails, n1!=n2 for Mann Whitney U test and Wilcoxon signed-rank test) and the effect size is set as 0.8.
Data exclusions	No data were excluded from the analyses.
Replication	We have verify the reproducibility of experimental findings. These results were verified by the human (n>10), animal (n=5-8/group) and cell (n>3) studies involved in the manuscripts.
Randomization	Human samples were allocated into groups according to clinical diagnostic criteria. Animals were grouped randomly. Detailed definitions and descriptions were provided in the manuscript.
Blinding	The investigators were blinded to the group allocation during data collection and the instrument running order was randomized. Investigators responsible for data analysis and manuscript preparation know the group allocation.

Reporting for specific materials, systems and methods

We require information from authors about some types of materials, experimental systems and methods used in many studies. Here, indicate whether each material, system or method listed is relevant to your study. If you are not sure if a list item applies to your research, read the appropriate section before selecting a response.

Materials & experimental systems

n/a	Involved in the study	
☐	☒	Antibodies
☐	☒	Eukaryotic cell lines
☒	☐	Palaeontology
☐	☒	Animals and other organisms
☐	☒	Human research participants
☒	☐	Clinical data

Methods

n/a	Involved in the study	
☒	☐	ChIP-seq
☒	☐	Flow cytometry
☒	☐	MRI-based neuroimaging

Antibodies

Antibodies used	FXR (Abcam, ab28480), FXR (Biorbyt, orb156973), FGF15 (Santa Cruz, sc398338), FGF19 (Abcam, ab85042), SHP (Abcam, ab186874), CYP7A1 (Abcam, ab65596), CYP8B1 (Abcam, ab191910), CYP27A1 (Abcam, ab126785), CYP7B1 (Abcam, ab138497), β-Actin (CST, 4970S), Lamin B1 (Beyotime, AF1408), anti-mouse IgG (CST, 7076S), anti-rabbit IgG (CST, 7074S).
Validation	The antibodies used in our study are for western-blot (WB) and immunofluorescence (IF) staining. The quality of the antibodies are all guaranteed by the manufacturers, and we further validated the antibody in varied assays for WB and IF detection.

Eukaryotic cell lines

Policy information about cell lines

Cell line source(s)	Human intestine FHs 74 Int cell line was purchased from American Type Culture Collection (ATCC). Human liver L02 cell line was purchased from Type Culture Collection of Chinese Academy of Science
Authentication	The authentication is guaranteed by ATCC and Type Culture Collection of Chinese Academy of Science
Mycoplasma contamination	The cell lines were tested negative for mycoplasma contamination.
Commonly misidentified lines (See ICLAC register)	No misidentified lines were used in this study.

Animals and other organisms

Policy information about studies involving animals; ARRIVE guidelines recommended for reporting animal research

Laboratory animals	C57BL/6J male mice at the age of four weeks were involved in this study.
Wild animals	The study did not involve wild animals.
Field-collected samples	All the mice were maintained in a specific-pathogen-free (SPF) environment with controlled conditions of a 12 h light/dark cycle at 20-22 °C and 45 ± 5% humidity.
Ethics oversight	All animal procedures were performed according to the national legislation and local guidelines of the Laboratory Animals Center at Shanghai Jiao Tong University, Shanghai, China.

Note that full information on the approval of the study protocol must also be provided in the manuscript.

Human research participants

Policy information about studies involving human research participants

Population characteristics	Thirteen healthy male volunteers at the age ranged from 24 to 32 years old were enrolled in this study.
Recruitment	Written informed consent was obtained from all the subjects before study initiation.All volunteers were provided with standard meals three times a day for a week before tea intervention and subsequently received 300 mL of tea infusion at 8:00 and 20:00 after standard meals for 4weeks at a dose of 50 mg/Kg/day. No other diets or drinks were consumed during the experimental period.
Ethics oversight	This study was conducted in accordance with the established ethical guidelines and approved by the research ethics committee of the School of Pharmacy, Shanghai Jiao Tong University, Shanghai, China.

Note that full information on the approval of the study protocol must also be provided in the manuscript.
