# Supplementary figures and images for: Theabrownin from Pu-erh tea attenuates hypercholesterolemia via modulation of gut microbiota and bile acid metabolism
Source: Nat Commun. 2019 Oct 31;10:4971. doi: 10.1038/s41467-019-12896-x (PMC6823360; doi:10.1038/s41467-019-12896-x)

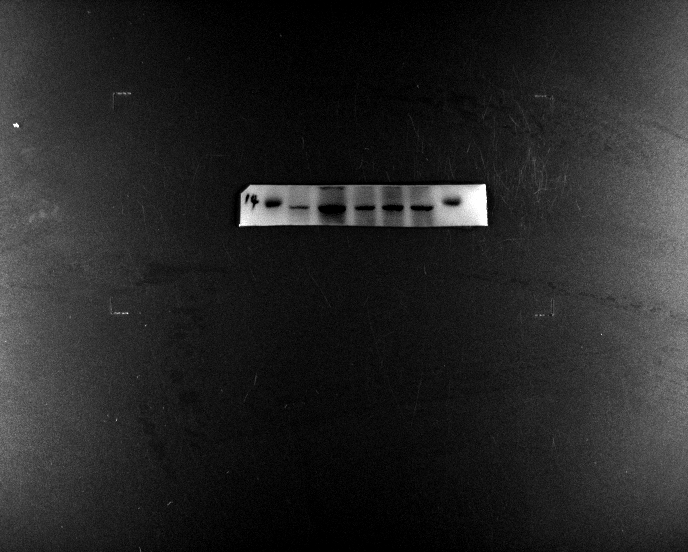

Supplement: Supplementary file 3 — Source Data [file 41467_2019_12896_MOESM3_ESM.zip › Source data/Blots/1-Fig 7d FHs 74 Int Nuclear-FXR.tif]

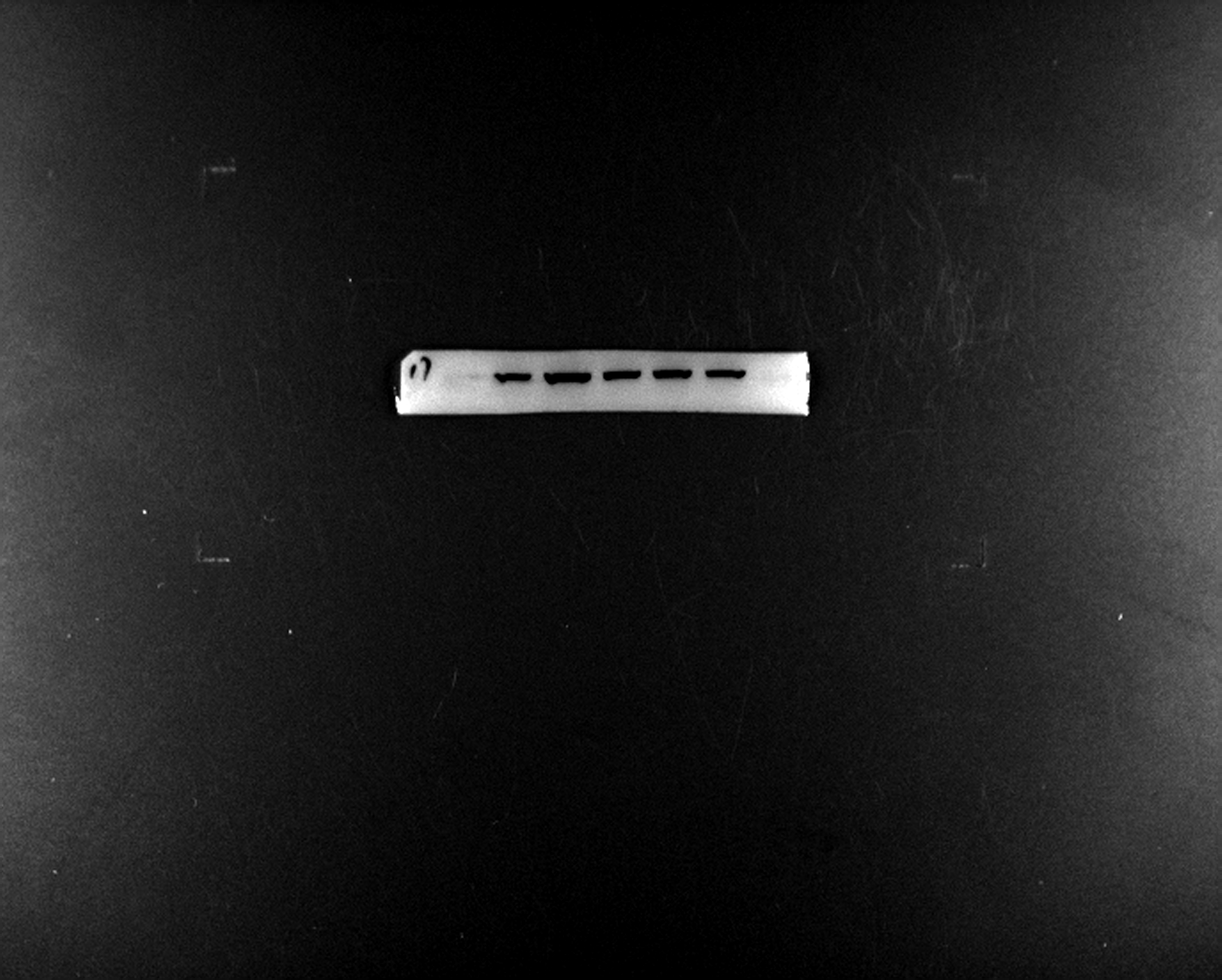

Supplement: Supplementary file 3 — Source Data [file 41467_2019_12896_MOESM3_ESM.zip › Source data/Blots/2-Lamin for nuclear FXR.tif]

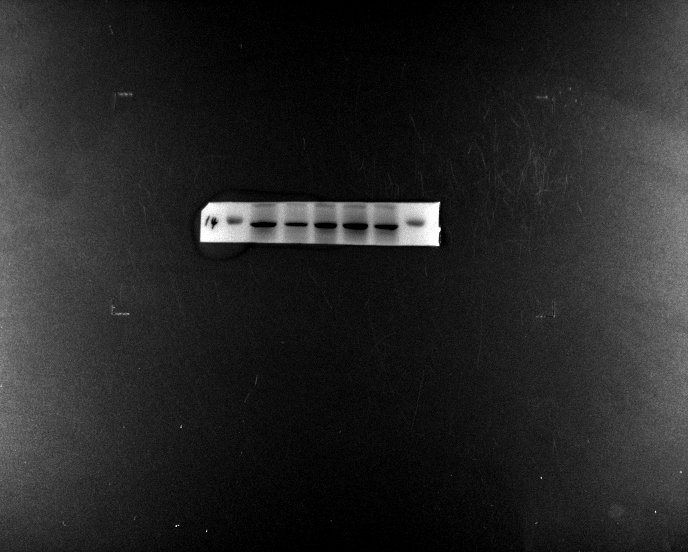

Supplement: Supplementary file 3 — Source Data [file 41467_2019_12896_MOESM3_ESM.zip › Source data/Blots/3-Fig 7d FHs 74 Int Cytoplasmic-FXR.tif]

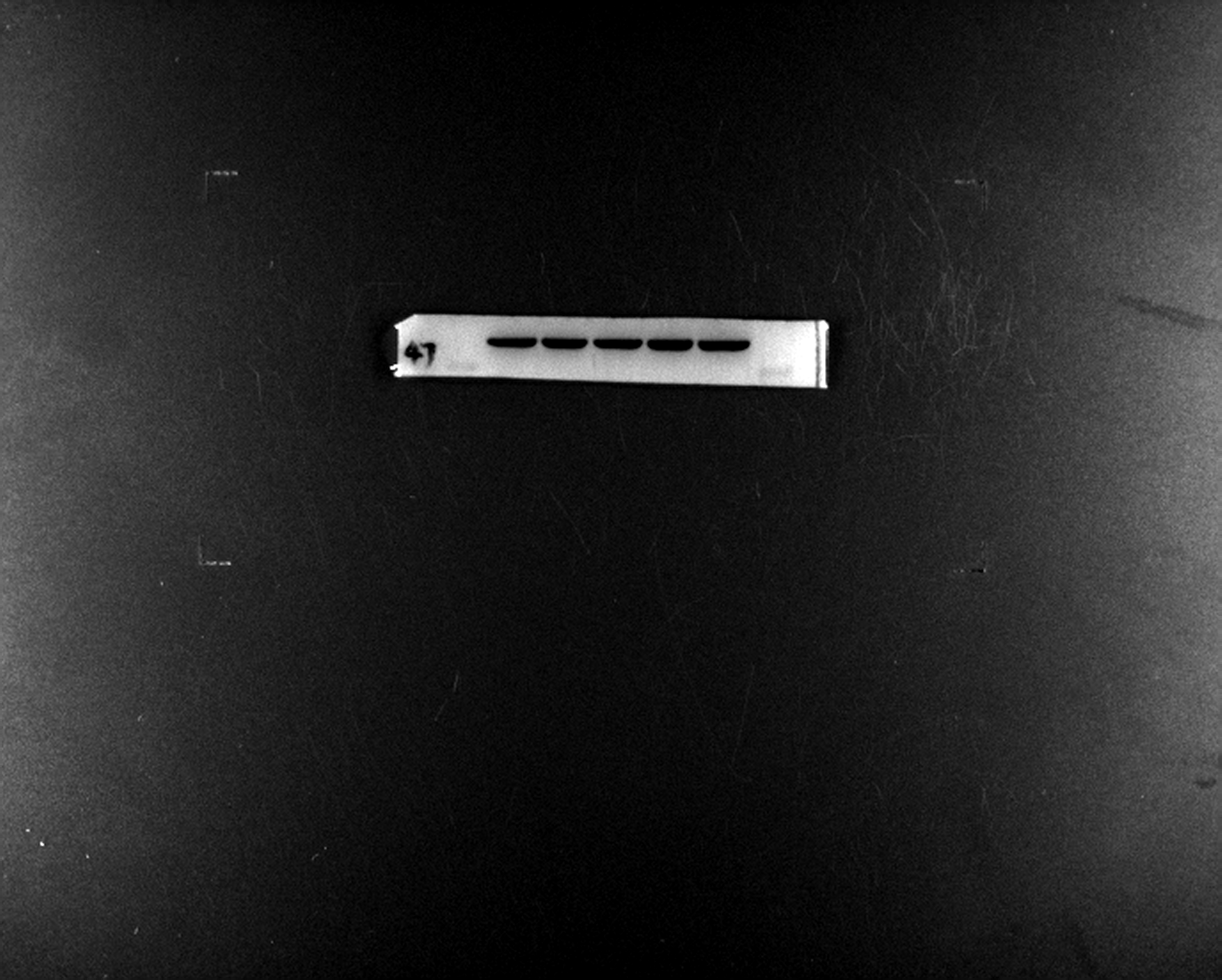

Supplement: Supplementary file 3 — Source Data [file 41467_2019_12896_MOESM3_ESM.zip › Source data/Blots/4-actin for cytoplasmic FXR.tif]

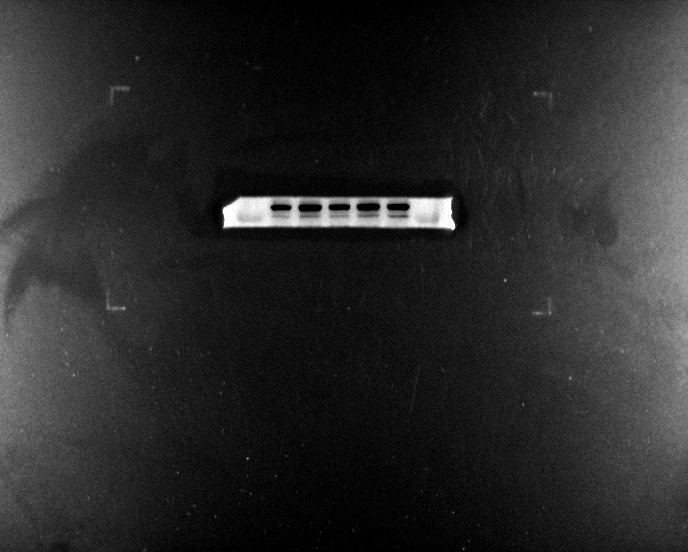

Supplement: Supplementary file 3 — Source Data [file 41467_2019_12896_MOESM3_ESM.zip › Source data/Blots/5-Fig 7d L02 Nuclear-FXR.tif]

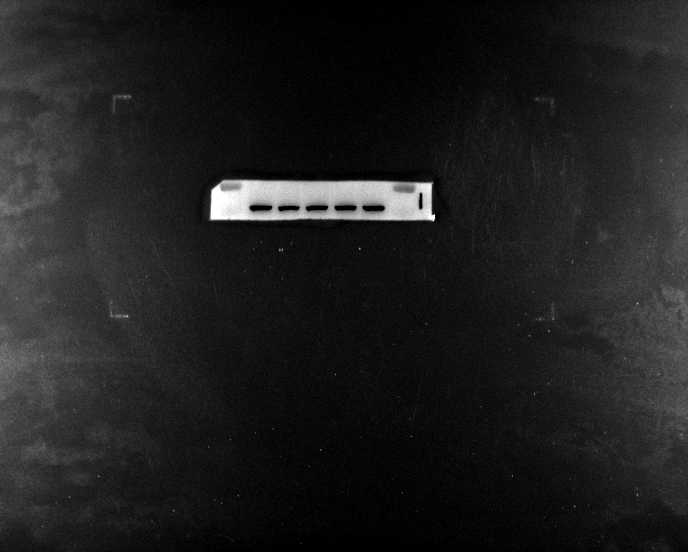

Supplement: Supplementary file 3 — Source Data [file 41467_2019_12896_MOESM3_ESM.zip › Source data/Blots/6-lamin for FXR.tif]

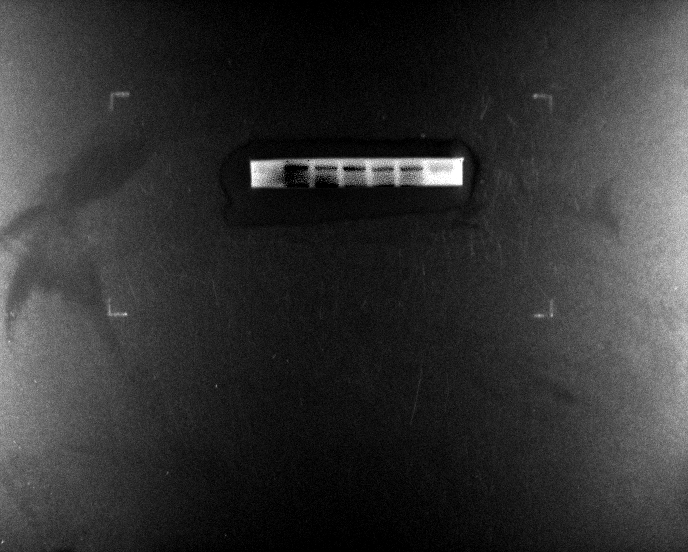

Supplement: Supplementary file 3 — Source Data [file 41467_2019_12896_MOESM3_ESM.zip › Source data/Blots/7-Fig 7d L02 Cytoplasmic-FXR.tif]

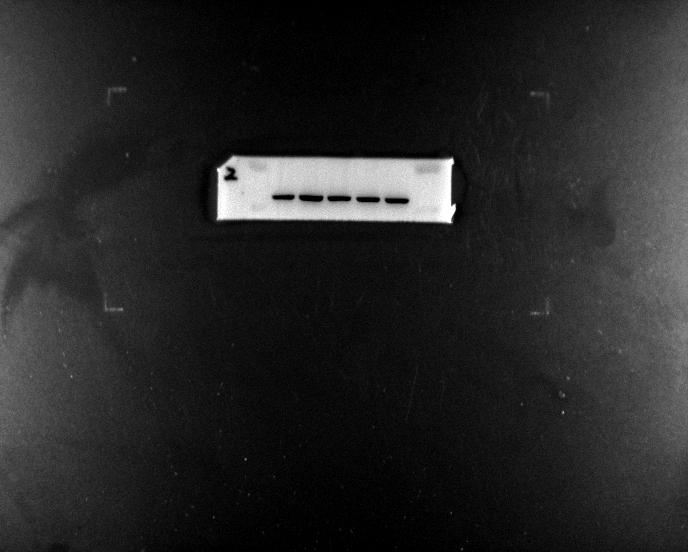

Supplement: Supplementary file 3 — Source Data [file 41467_2019_12896_MOESM3_ESM.zip › Source data/Blots/8-actin for FXR.tif]
